# Caspase 6 promotes innate immune activation by functional crosstalk between RIPK1-IκBα axis in liver inflammation

**DOI:** 10.1186/s12964-023-01287-x

**Published:** 2023-10-12

**Authors:** Yuanbang Lin, Mingwei Sheng, Hua Qin, Peng Zhang, Chunli Wang, Wei Fu, Xiangjun Meng, Duowei Wang, Yachao Hou

**Affiliations:** 1 Department of General Surgery, Tianjin Medical University General Hospital, Anshan Road NO. 154, Tianjin, 300052 PR China China; 2 Department of Anesthesiology, Tianjin First Central Hospital, Tianjin, China; 3 College of Life Sciences, Nankai University, Tianjin, 300071 China

**Keywords:** Liver Ischemia reperfusion, Innate immunity, Ferroptosis, Caspase 6

## Abstract

**Background:**

Caspase 6 is an essential regulator in innate immunity, inflammasome activation and host defense. We aimed to characterize the causal mechanism of Caspase 6 in liver sterile inflammatory injury.

**Methods:**

Human liver tissues were harvested from patients undergoing ischemia-related hepatectomy to evaluate Caspase 6 expression. Subsequently, we created Caspase 6-knockout (Caspase 6^KO^) mice to analyze roles and molecular mechanisms of macrophage Caspase 6 in murine models of liver ischemia/reperfusion (IR) injury.

**Results:**

In human liver biopsies, Caspase 6 expression was positively correlated with more severe histopathological injury and higher serum ALT/AST level at one day postoperatively. Moreover, Caspase 6 was mainly elevated in macrophages but not hepatocytes in ischemic livers. Unlike in controls, the Caspase 6-deficient livers were protected against IR injury, as evidenced by inhibition of inflammation, oxidative stress and iron overload. Disruption of macrophage NF-κB essential modulator (NEMO) in Caspase 6-deficient livers deteriorated liver inflammation and ferroptosis. Mechanistically, Caspase 6 deficiency spurred NEMO-mediated IκBα phosphorylation in macrophage. Then phosphorylated-inhibitor of NF-κBα (p-IκBα) co-localized with receptor-interacting serine/ threonine-protein kinase 1 (RIPK1) in the cytoplasm to degradate RIPK1 under inflammatory conditions. The disruption of RIPK1-IκBα interaction preserved RIPK1 degradation, triggering downstream apoptosis signal-regulating kinase 1 (ASK1) phosphorylation and inciting NIMA-related kinase 7/NOD-like receptor family pyrin domain containing 3 (NEK7/NLRP3) activation in macrophages. Moreover, ablation of macrophage RIPK1 or ASK1 diminished NEK7/NLRP3-driven inflammatory response and dampened hepatocyte ferroptosis by reducing HMGB1 release from macrophages.

**Conclusions:**

Our findings underscore a novel mechanism of Caspase 6 mediated RIPK1-IκBα interaction in regulating macrophage NEK7/NLRP3 function and hepatocytes ferroptosis, which provides therapeutic targets for clinical liver IR injury.

**Graphical Abstract:**

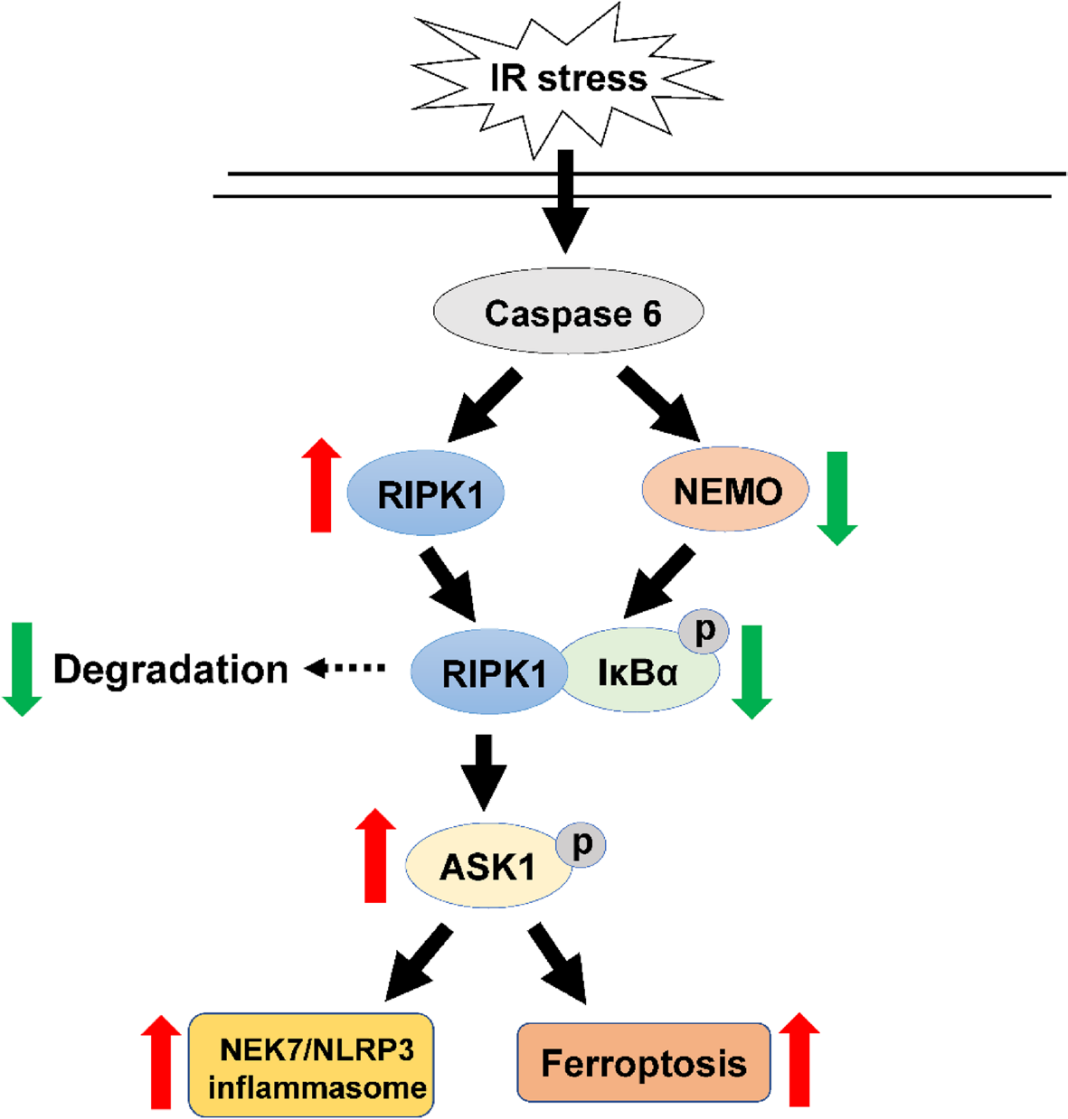

Video Abstract

**Supplementary Information:**

The online version contains supplementary material available at 10.1186/s12964-023-01287-x.

## Background

Liver ischemia reperfusion (IR) injury is an inevitable complication during hepatectomy and liver transplantation [1]. Recent years have witnessed rapid progress in comprehending the mechanisms of liver IR, including calcium overload, release of proinflammatory mediators, and lipid metabolism disturbance [2]. In the initial stage of hepatic IR, macrophage activation can incite the release of various danger associated molecular patterns (DAMPs) including high mobility group protein 1 (HMGB1) from injured or dead cells, triggering intense innate immune responses [3, 4], which are identified to be one of the leading causes contributing to liver dysfunction or graft rejection after liver transplantation.

Cysteinyl aspartate specific proteinase (Caspase) is a family of enzymes that regulates programmed cell death, inflammation and other biological functions [5]. To date, 14 identified caspases are functionally grouped as apoptosis initiators (Caspase 2, Caspase 8, etc.), apoptosis executors (Caspase 1, Caspase 6, etc.) and inflammatory regulators (Caspase 4, Caspase 5, etc.). Although Caspase 6 was first identified as an executor of apoptosis, its activation and cleavage mechanisms remain poorly understood. In recent years, emerging evidence suggests that its function is not limited to regulate apoptosis, but also to the progression of neurodegenerative diseases such as Alzheimer's disease and Huntington's disease [6]. In nonalcoholic steatohepatitis model, the AMP-activated protein kinase (AMPK)-Caspase 6 axis generated a feedforward loop to regulate hepatocyte death and liver damage [7]. An increasing number of studies have demonstrated that Caspase 6 orchestrates cell death under various ischemia-related conditions [8, 9]. Additionally, Caspase 6 knockdown was reported to increase retinal ganglion cell survival and decrease the neuropathological consequences in ischemic neuronal degeneration [10]. Our previous study also illustrated the pivotal role of Caspase 6-induced inflammasome activation in ischemia-stressed fatty liver [11]. Considering the specific function of Caspase 6 in liver parenchymal (hepatocytes) and immune cells (macrophages, neutrophils etc.), there are crucial knowledge gaps concerning cell-specific Caspase 6 functions in controlling innate immune-driven sterile hepatic inflammation. A deeper understanding of Caspase 6 mediated macrophage-hepatocytes crosstalk in the context of hepatic IR-related insult is worthy of investigation.

Receptor-interacting serine/threonine-protein kinase 1 (RIPK1) is integral in regulating innate immune signaling and inflammatory responses [12], and is considered one of the promising therapeutic targets for multiple diseases. Cell death and inflammation pathways are carefully orchestrated by reciprocal modification of RIPK1 [13]. TGFβ activated kinase 1 (TAK1) Knockout in macrophages can drive an increase in RIPK1 kinase activity to mediate programmed cell death and systemic inflammatory responses [14]. Moreover, Caspase 8 inhibited apoptosis and programmed necrosis through the cleavage of RIPK1 [15]. Highly-selective small-molecule inhibitors of RIPK1 based on its unique hydrophobic pocket in the allosteric regulatory domain have demonstrated safety and efficacy in preclinical models and clinical trials [16]. However, the potential regulation of RIPK1 by Caspase 6 in liver IR model is virtually unexplored.

Herein, we hypothesize that Caspase 6 is a vital component for activation of innate immune responses during liver IR stress. First, hepatic Caspase 6 expression and liver function were determined in patients undergoing hepatectomy and mice liver IR model. Second, Caspase 6-knockout mice were introduced to investigate the functional role of Caspase 6 in innate immunity induced by liver IR stimulation. Third, the precise mechanism of Caspase 6 in regulating programmed cell death and inflammasome activation in macrophages was deeply explored in vivo and in vitro.

## Materials & methods

### Patients and clinical samples

This study was approved by the Academic Committee of Tianjin Medical University General Hospital. Human samples were obtained from 30 patients with benign liver tumors who underwent partial hepatectomy with pringle maneuver (January 2021-December 2021, Department of General Surgery, Tianjin Medical University General Hospital, patient characteristics were shown in Supplementary Table 1). Liver biopsies were acquired after reperfusion during hepatectomy (before abdominal closure, ischemic time, 15–30 min; reperfusion time, 15-20 min). Hepatocyte damage was assessed by detecting serum alanine aminotransferase (ALT) and aspartate transaminase (AST) in the first 1–7 days postoperatively (POD1-7). Informed consent was obtained from all objects.

### Mice liver warm IR model

Caspase 6-knockout (KO) C57BL/6N mice were generated by CRISPR/Cas9 technology (Cyagen Biosciences, Jiangsu, China). All protocols of animal experiments were approved by the Animal Ethics Committee of Tianjin First Central Hospital. Male 6–8 weeks old wild-type (WT) mice or Caspase 6^KO^ mice were used to establish mouse liver IR model as described in our previous studies [17]. Briefly, arterial/portal vessels were clipped using an atraumatic vascular clip for 90 min. Then the clip was released to initiate reperfusion. Liver tissue or serum was harvested at 6 h of reperfusion. The same procedure was performed in the Sham group, but without vessels occlusion. AlexaFluor488-labeled non-specific (NS) siRNA or NEMO siRNA (1 mg/kg; Santa Cruz Biotechnology, CA) mixed with mannose-conjugated polymers (Polyplus transfection™, Illkirch, France) were injected through the tail vein of mice 24 h prior to IR surgery [4].

### ALT/AST detection

Serum ALT and AST levels were detected using the ALT kit and AST kit (Sigma Aldrich), according to the manufacturer’s instructions.

### ELISA assay

Serum or culture medium LDH, IL-1β and HMGB1 were detected using commercial ELISA kits (ThermoFisher).

### Iron content detection

The iron content of liver tissues was detected using Iron assay kit (ab83366, Abcam). Briefly, tissue extracts were homogenized in iron assay buffer on ice then centrifuged at 16000 g for 10 min at 4 °C. The supernatant was transferred and incubated with assay buffer. The mixture was incubated at 37 °C for 30 min followed by incubation with iron probe at 37 °C for 30 min protected from light. Output was measured at 593 nm with a colorimetric microplate reader.

### MDA detection assay

Liver tissues or cultured cells were homogenated and subjected to Malonydialdehyde (MDA) detection assay according to instructions of MDA assay kit (S0131, Beyotime).

### Histopathology

Livers were fixed in 4% paraformaldehyde for 24 h, subsequently embedded in paraffin and sectioned into 5 μm-thick slices. For liver histopathology, sections underwent hematoxylin–eosin staining. Suzuki's criteria were applied to assess the histological severity of liver damage. For immunohistochemistry (IHC) analysis, liver sections were first rehydrated and processed for antigen exposure, followed by incubation with IL1β (Abcam, ab283818, 1:100 dilution), Ly6G (Abcam, ab261916, 1:100 dilution), 4-HNE (Abcam, ab48506, 1:100 dilution) or ACSL4 (Abcam, ab205199, 1:100 dilution) primary antibodies respectively at 4 °C overnight. For immunofluorescence staining, tissue sections or cultured cells were fixed in 4% paraformaldehyde for 15 min, incubated overnight at 4 °C with primary antibodies including CD11b (Abcam, ab184308, 1:100 dilution), CD68 (Cell signaling Technology, #26,042, 1:100 dilution), IκBα (Cell signaling Technology, #4814, 1:100 dilution), RIPK1 (Cell signaling Technology, #3493, 1:100 dilution), NLRP3 (Abcam, ab270449, 1:100 dilution) or GPX4 (Abcam, ab125066, 1:100 dilution) respectively. The secondary antibodies were conjugated with AlexaFlour 488 or AlexaFlour Cy5 (Jackson Immunoresearch), protected from light at room temperature for 2 h. Immunofluorescence images were captured using a fluorescence microscope (Keyence BZ-X810, Osaka, Japan). The positive-stained cells were calculated in three representative high-power fields (HPF) from each tissue section.

### Quantitative RT-PCR analysis

Total RNA was extracted using Trizol reagent (15,596,026, Invitrogen). Reverse was transcribed into cDNA according to the instructions of PrimeScript RT kit (A15300, Invitrogen). qPCR was carried out using SYBR Green PCR Kit (4,367,659, Applied biosystems). β-actin served as internal control. The primers (Caspase 6, IL-1β, TNF-α, CXCL-10, β-actin) were listed in Supplementary Table 2.

### Western blot analysis

Protein extracts was prepared in RIPA lysis buffer (89,900, ThermoFisher) and homogenized at 4 °C for 30 min. Supernatant was collected by centrifugation at 12000 g for 30 min. Proteins (40 μg/sample) were separated by SDS–polyacrylamide gel electrophoresis (PAGE) and transfer to nitrocellulose membrane. Western blot assay was performed with antibodies including Caspase 6 (Cell signaling Technology, #9762, 1:100 dilution), NEMO (Cell signaling Technology, #2685, 1:1000 dilution), IκBα (Cell signaling Technology, #4812, 1:1000 dilution), p-IκBα (Cell signaling Technology, #2859, 1:1000 dilution), NEK7 (Cell signaling Technology, #10,054, 1:1000 dilution), NLRP3 (Abcam, ab270449, 1:1000 dilution), Cleaved-caspase 1 (Cell signaling Technology, #89,332, 1:1000 dilution), RIPK1 (Cell signaling Technology, #3493, 1:1000 dilution), NOX1 (Abcam, ab131088, 1:1000 dilution), β-actin (Cell signaling Technology, #4970, 1:2000 dilution), p-ASK1 (Cell signaling Technology, #3765, 1:250 dilution), GPX4 (Abcam, ab125066, 1:1000 dilution), ACSL4 (Abcam, ab155282, 1:1000 dilution), HMGB1 (Cell signaling Technology, #3935, 1:1000 dilution), anti-rabbit IgG (Cell signaling Technology, #7074, 1:2000 dilution), and anti-mouse IgG (Cell signaling Technology, #7076, 1:2000 dilution). IBright FL1000 (Invitrogen) was used to qualify the expression of proteins and β-actin served as the control.

### Primary isolation of hepatocytes and Kupffer cells

Primary hepatocytes and Kupffer cells were isolated according to the method as described [4]. In general, mouse liver was digested in situ with 1 mmol/L EGTA pre-warmed at 37 °C followed by 0.75 g/L type I collagenase solution pre-warmed at 37 °C. Live hepatocytes were separated by centrifugation then seeded onto 6 or 12-well plates. Nonparenchymal cells (NPCs) were separated from hepatocytes by centrifugation at 50 g for 2 min. NPCs were resuspended in HBSS and separated using 50%/25% two-step Percoll gradient (1800 g at 4 °C for 15 min). Kupffer cells, located in the centrifuge tube's middle layer, were harvested and resuspended in DMEM culture medium, and the non-adherent cells were removed by medium exchange.

### BMMs isolation and in vitro transfection

Murine bone-derived macrophages (BMMs) were isolated from mice femur and tibia, then filtered through a 200 μm nylon mesh cell strainer (BD Biosciences). After centrifugation at 300 g for 10 min, cell pellet was resuspended in 15% L929-conditioned medium supplemented with 10% FBS. Cells (1 × 10^6^/well) were cultured for 7 days and then transfected with NEMO siRNA, CRISPR-RIPK1 activation, CRISPR-ASK1 activation, CRISPR/Cas9-RIPK1 KO, CRISPR/Cas9-ASK1 KO or control vector (Santa Cruz Biotechnology). After 24-48 h, they were treated with or without LPS (100 ng/ml) for 6 h.

### Co-culture of macrophages and hepatocytes

BMMs (1 × 10^6^/well) were added to a 0.4 μm-pore size Transwell chamber and transfected with the CRISPR-ASK1 Activation, KO or control vector followed by LPS stimulation. Primary hepatocytes (5 × 10^5^/well) were incubated in 6-well plate for 24 h. Then the Transwell chambers containing BMMs was inserted into the 6-well plate seeded with hepatocytes. Co-cultures were incubated for 24 h with or without 200 µM H_2_O_2_.

### Co-immunoprecipitation analysis

Cells were lysed in NP-40 lysis buffer (50 mM Tris pH7.4, 10 mM EDTA, 150 mM NaCl, 1% NP-40). Total cell extracts were incubated with RIPK1 (Cell signaling Technology, #3493) or IκBα (Cell signaling Technology, #4812) antibody respectively at 4 °C overnight, followed by incubation with protein-G/A beads at 4 °C for 4 h. the pellet was harvested by centrifugation and then eluted by heating at 95 °C for 5 min. The supernatant was collected and analyzed by standard immunoblot procedures.

### Statistical analysis

SPSS 22.0 software was used for analysis. All data were expressed as the mean ± standard deviation (SD). The statistical comparison between multiple groups was carried out by one-way analysis of variance (ANOVA) or student`s t-test. Linear regression (R2) was used to analyze the strength of linear relationship between variables. P < 0.05 was considered statistically significant.

## Results

### IR induced Caspase 6 expression correlates positively with liver injury in human and mice biopsies

**Fig. 1:**
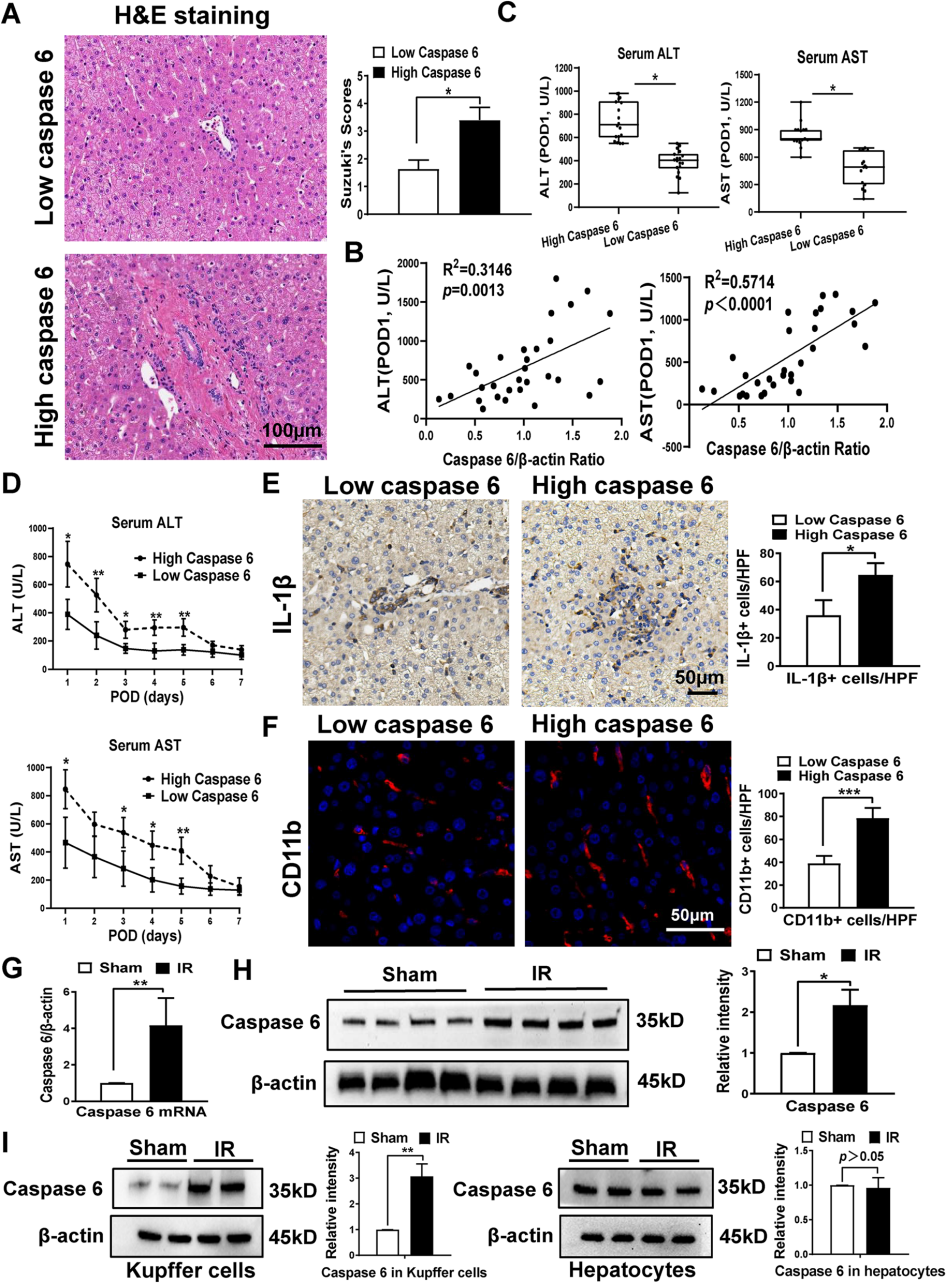
IR induced Caspase 6 expression correlates positively with liver injury in human and mice biopsies. Liver samples were harvested from patients during hepatectomy (after hepatic portal vein occlusion). **A** H&E staining and Suzuki’s score in High Caspase 6 and Low Caspase 6 groups using the median Caspase 6/β-actin as cutoff value, scale bar: 100 µm, *N* = 5/group. **B** Serum ALT/AST was positively correlated with Caspase 6/β-actin by qRT-PCR assay at the first day postoperatively (POD1), *N* = 15/group. **C** The level of ALT/AST in High Caspase 6 and Low Caspase 6 groups, *N* = 15/group. **D** Serum ALT/AST levels of patients in 7 days after hepatectomy, *N* = 15/group. **E**–**F** IL-1β IHC staining and CD11b IF staining in human liver biopsies, scale bar: 50 µm, N = 5/group. **G** qRT-PCR analysis of Caspase 6 in livers induced by IR, *N* = 5/group. **H** WB analysis of Caspase 6 in mice ischemic livers, *N* = 4/group. **I** WB-assisted Caspase 6 expression profile in Kupffer cells and hepatocytes isolated from mice ischemic livers, *N* = 6/group. All data represent the mean ± SD, *p < 0.05, **p < 0.01

To explore the role of Caspase 6 in the pathogenesis in liver IR, Caspase 6 expression profile was firstly evaluated by qRT-PCR assay in the liver biopsies from patients undergoing partial hepatectomy. Ischemia time (hepatic portal vein occlusion time) was 15-30 min and reperfusion time was 15-20 min. Subsequently, these individuals were categorized into Low-Caspase 6 and High-Caspase 6 groups using the median Caspase 6/β-actin ratio as cutoff value (1.01). Pathological examinations revealed aggravated vacuolization, sinusoidal congestion in High-Caspase 6 expressed liver tissues (Fig. 1A). Furthermore, liver Caspase 6 expression held a positive correlation with serum ALT and AST levels at POD1 (Fig. 1B). Patients characterized by higher Caspase 6 expression exhibited increased ALT and AST levels at POD1, alongside delayed liver function recovery within 7 days postoperatively (Fig. 1C-D). This suggests that IR-triggered Caspase 6 expression may facilitate liver injury. Moreover, increased IL-1β secretion and macrophage activation in High-Caspase 6 group (Fig. 1E-F). Similarly, liver IR amplified the mRNA and protein levels of Caspase 6 in mice models (Fig. 1G-H). In order to figure out the cell-specific expression of Caspase 6, we isolated hepatocytes and liver macrophages (Kupffer cells) from ischemic livers. Western blotting revealed that IR primarily triggered the activation of Caspase 6 in macrophages but not hepatocytes (Fig. 1I).

### Disruption of Caspase 6 alleviates IR-induced liver injury, macrophage/neutrophil infiltration, inflammation, oxidative stress and iron overload in IR-stressed livers

**Fig. 2:**
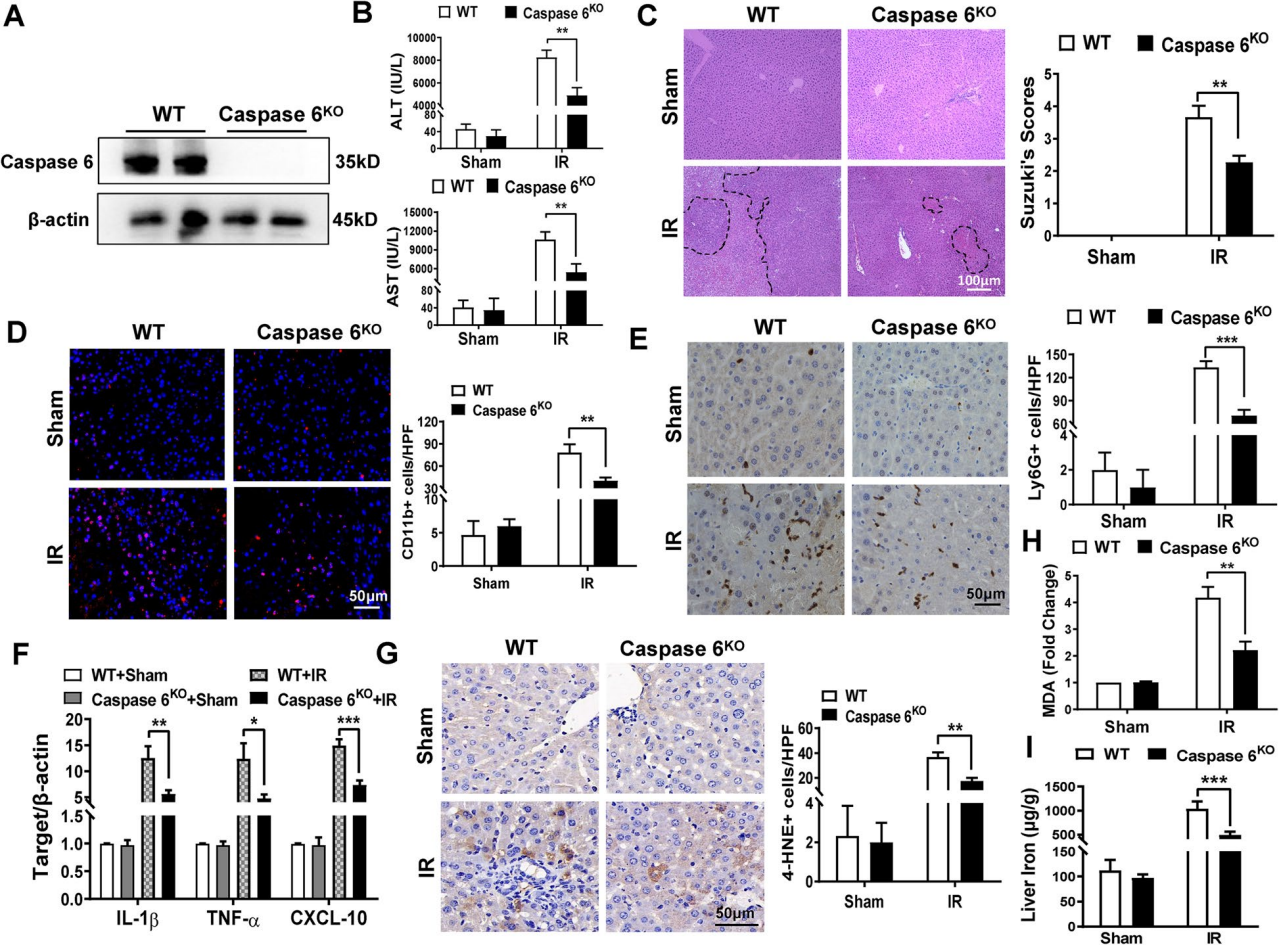
Disruption of Caspase 6 alleviates IR-induced liver injury, and reduces macrophage/neutrophil infiltration, inflammation, oxidative stress and iron overload in IR-stressed livers. WT and Caspase 6.^KO^ mice were used to establish liver IR models. Samples were harvested after 90 min ischemia and 6 h of reperfusion, *N* = 6/group. **A** Liver Caspase 6 expression was evaluated by Western blot assay, *N* = 6/group. **B** Serum ALT/AST level was detected in ischemia livers; (**C**) Representative H&E staining and Suzuki’s score in ischemic liver tissue. scale bar: 100 μm, *N* = 6/group. **D** Immunofluorescence staining and quantification of CD11b + macrophages in ischemia livers, scale bar: 50 μm, *N* = 4/group. **E** IHC staining and quantification of Ly6G + neutrophiles in ischemia livers, scale bar: 50 μm, *N* = 4/group. **F** Detection of cytokines IL-1β, TNF-α and CXCL-10 by qRT-PCR in ischemic livers, *N* = 6/group. Lipid peroxidation by using 4-HNE staining, scale bar: 50 μm, *N* = 6/group (**G**) and colorimetric assay, *N* = 6/group (**H**); (**I**) Non-heme iron profiles in liver tissues were measured by colorimetric assay, *N* = 6/group. All data represent the mean ± SD, *p < 0.05, **p < 0.01, ***p < 0.001

To better clarify the functional role of Caspase 6 in IR induced liver inflammatory and oxidative injury, we utilized CRISPR/Cas9 system to create Caspase 6-knockout strain (Caspase 6^KO^). Indeed, compared with WT group, the total expression of Caspase 6 was not detected in livers from Caspase 6^KO^ mice (Fig. 2A). After ischemia 90 min followed by reperfusion 6 h, serum ALT/AST level was reduced in Caspase 6^KO^ mice compared to WT controls (Fig. 2B). Furthermore, Caspase 6^KO^ mice exhibited mild to moderate hepatocytes necrosis, edema and sinusoidal congestion (Fig. 2C). The accumulation of CD11b + macrophages (Fig. 2D) and Ly6G + neutrophils (Fig. 2E) were suppressed in Caspase 6^KO^ ischemic livers, accompanied by down-regulated mRNA expressions coding for IL-1β, TNF-α and CXCL-10 (Fig. 2F). Oxidative stress was inhibited as evidenced by dampened lipid peroxidation by using 4-HNE staining (Fig. 2G) and colorimetric assay (Fig. 2H). Interestingly, iron accumulation was also inhibited in Caspase 6^KO^ ischemic livers (Fig. 2I).

### Caspase 6 deficiency regulates RIPK1/IκBα signaling and inhibits NEK7/NLRP3 activation and ferroptosis in IR-stressed livers

**Fig. 3:**
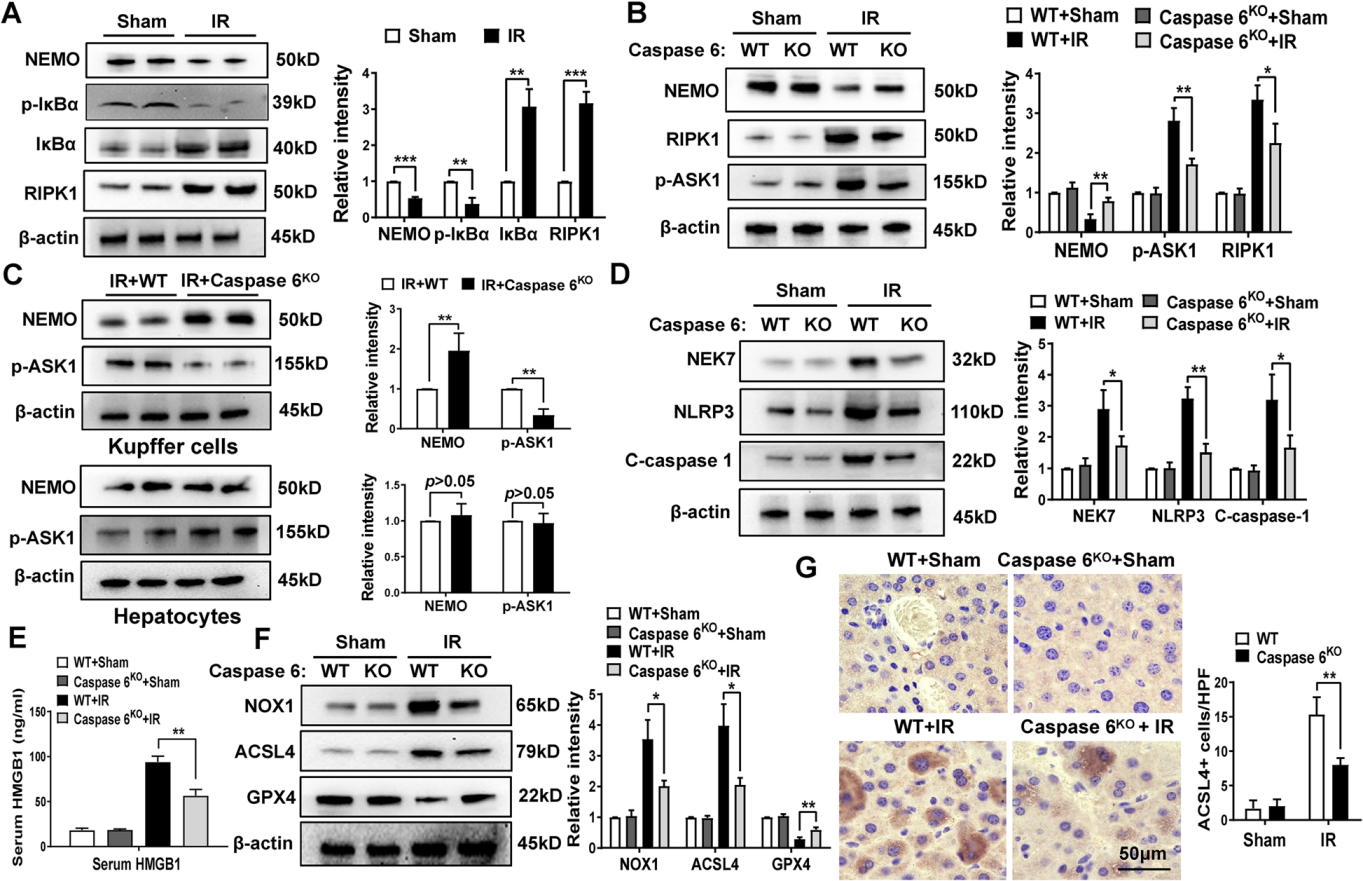
Disruption of Caspase 6 regulates RIPK1/IκBα signaling and inhibits NEK7/NLRP3 activation and ferroptosis in IR-stressed livers. Western-assisted analysis and relative density ratio of (**A**) NEMO, p-IκBα, IκBα, RIPK1 in Sham and IR groups; (**B**) RIPK1, NEMO, p-ASK1 in the WT and Caspase 6^KO^ mice after liver IR; (**C**) NEMO and p-ASK1 in Kupffer cells and hepatocytes isolated from ischemic livers; (**D**) NEK7, NLRP3, and cleaved caspase-1 in the ischemic livers, *N* = 6/group. **E** ELISA assay of serum HMGB1 in the WT and Caspase 6^KO^ mice after liver IR, *N* = 6/group. **F** The expression and relative intensity of NOX1, ACSL4 and GPX4 in IR-induced WT and Caspase 6^KO^ livers, *N* = 6/group. **G** IHC staining of ACSL4 expression in IR-induced livers, scale bar: 50 μm, *N* = 4/group. All data represent the mean ± SD, *p < 0.05, **p < 0.01, ***p < 0.001

Given that the activation of NEMO or RIPK1 mediated innate immunity response initiates liver inflammation, we analyzed the effects of Caspase 6 on the inflammatory program in vivo. We found that the NEMO signaling was curtailed, as evidenced by the reduced expression of NEMO and p-IκBα, accompanied by less degradation of total IκBα. IR also induced RIPK1 expression (Fig. 3A). Moreover, Caspase 6 deletion deterred the phosphorylation of RIPK1 and ASK1 while enhancing NEMO expression (Fig. 3B). Unlike in hepatocytes, Caspase 6 deficiency did escalate the levels of NEMO and ASK1 phosphorylation in macrophages (Fig. 3C) and dampened NEK7/NLRP3 inflammasome activation (Fig. 3D), suggesting a crucial role of Caspase 6 in macrophage NEMO signaling-mediated inflammatory response. Subsequently, we measured serum HMGB1 level, a critical mediator liberated from inflamed cells. Indeed, Caspase 6-deficiency resulted in a noticeable reduction of HMGB1 compared to WT + IR group (Fig. 3E). Interestingly, IR triggered increased NADPH oxidase 1 (NOX1), Acyl-CoA synthetase long-chain familymember4 (ACSL4) companied with reduced GPX4, which were the hallmarks in the progression of ferroptosis (Fig. 3F). Consistently, IHC staining reaffirmed the diminished level of ACSL4 in Caspase 6-deficient ischemic livers (Fig. 3G).

### NEMO is required for the regulation of NEK7/NLRP3 function, oxidative stress and iron overload in Caspase 6-deficient livers in response to IR injury

**Fig. 4:**
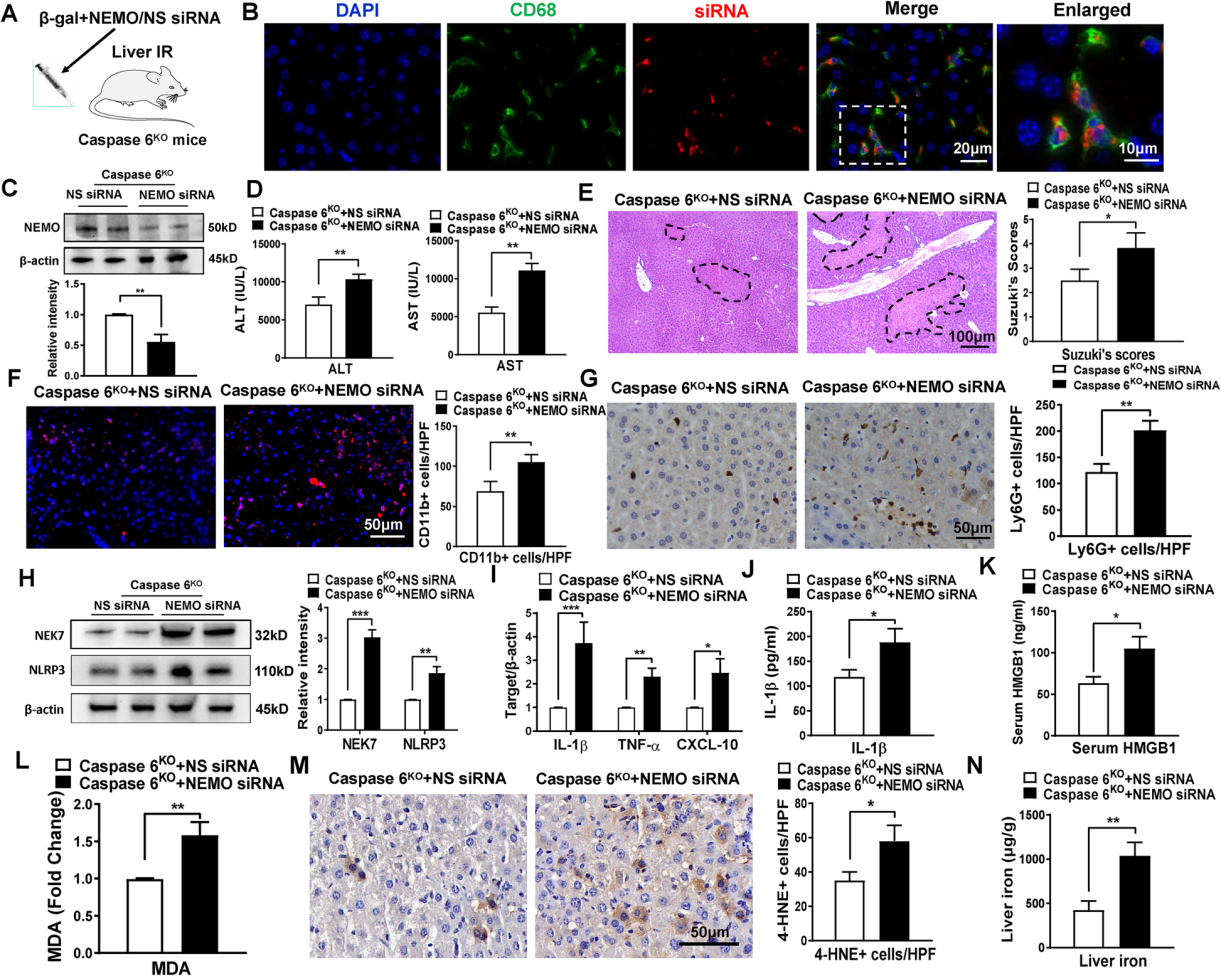
NEMO is required for the regulation of NEK7/NLRP3 function, oxidative stress and iron overload in Caspase 6-deficient livers in response to IR stress. **A** Schematic figure for the injection of mannose-conjugated NEMO siRNA delivery system into ischemic livers of Caspase 6^KO^ mice. **B** Immunofluorescence staining of AlexaFluor488-labeled CD68 positive macrophages and AlexaFluor Cy5-labeled control siRNA in ischemic livers, Scale bars, 20 μm, 10 μm, *N* = 4/group. **C** Liver NEMO expression was evaluated by Western blot assay, *N* = 6/group. **D** Serum ALT/AST level in ischemic livers, *N* = 6/group. **E** H&E staining of ischemic livers and Suzuki’s scores. Scale bar: 100 μm, *N* = 6/group. **F** Immunofluorescence staining and quantification of CD11b^+^ macrophages in ischemic livers, Scale bar: 50 μm, *N* = 4/group. **G** IHC staining and quantification of Ly6G.^+^ neutrophils in ischemic livers, Scale bar: 50 μm, *N* = 4/group. **H** Western blotting analysis and relative intensity of NEK7 and NLRP3, *N* = 6/group. **I** Detection of cytokines IL-1β, TNF-α and CXCL-10 by qRT-PCR in ischemic livers, *N* = 6/group. ELISA analysis of serum IL-1β levels (**J**) and serum HMGB1 levels (**K**), *N* = 6/group. **L** Hepatic MDA concentration in ischemic livers, *N* = 6/group. **M** 4-HNE staining in IR-induced liver tissues, scale bar: 50 μm, *N* = 4/group. **N** Non-heme iron profiles in liver tissues by colorimetric assay, *N* = 6/group. All data represent the mean ± SD, *p < 0.05, **p < 0.01, ***p < 0.001

As disruption of Caspase 6 activated the NEMO/IκBα pathway, we probed whether NEMO regulated NEK7/NLRP3 function, oxidative stress and iron overload in IR stressed livers. Consistent with previous reports by ours and others [4, 18], we disrupted NEMO in Caspase 6^KO^ livers using a mannose-conjugated NEMO siRNA delivery system. This system specifically delivered siRNA to macrophages via a mannose-specific membrane receptor (Fig. 4A). Indeed, mannose-mediated AlexaFluorCy5-labeled siRNA (red) delivery was efficiently transduced into macrophages (green) in IR-stressed livers (Fig. 4B), WB assay furthermore confirmed a significant decrease of NEMO in NEMO-siRNA pretreated livers (Fig. 4C). Compared to the NS siRNA controls, knockdown of macrophage NEMO in the Caspase 6^KO^ mice with the mannose-mediated siRNA treatment exacerbated functional and pathological liver damage. This was evident from the increased serum ALT/AST levels and Suzuki’s score (Fig. 4D-E). NEMO siRNA injection in the Caspase 6^KO^ ischemic livers upregulated accumulation of CD11b + macrophages (Fig. 4F) and Ly6G + neutrophils (Fig. 4G). Moreover, the reduced level of macrophage NEMO in Caspase 6^KO^ mice led to NEK7/NLRP3 inflammasome activation, as shown by the increased NEK7 and NLRP3 expression (Fig. 4H), up-regulated cytokine mRNA expression (Fig. 4I) and serum IL-1β liberation (Fig. 4J). The serum HMGB1 release (Fig. 4K), oxidative stress (Fig. 4L-M) and liver iron overload (Fig. 4N) were also augmented compared with the NS-siRNA-treated groups.

### NEMO mediated RIPK1-IκBα interaction induces RIPK1 degradation while inhibited NEK7/NLRP3 function in macrophages

**Fig. 5:**
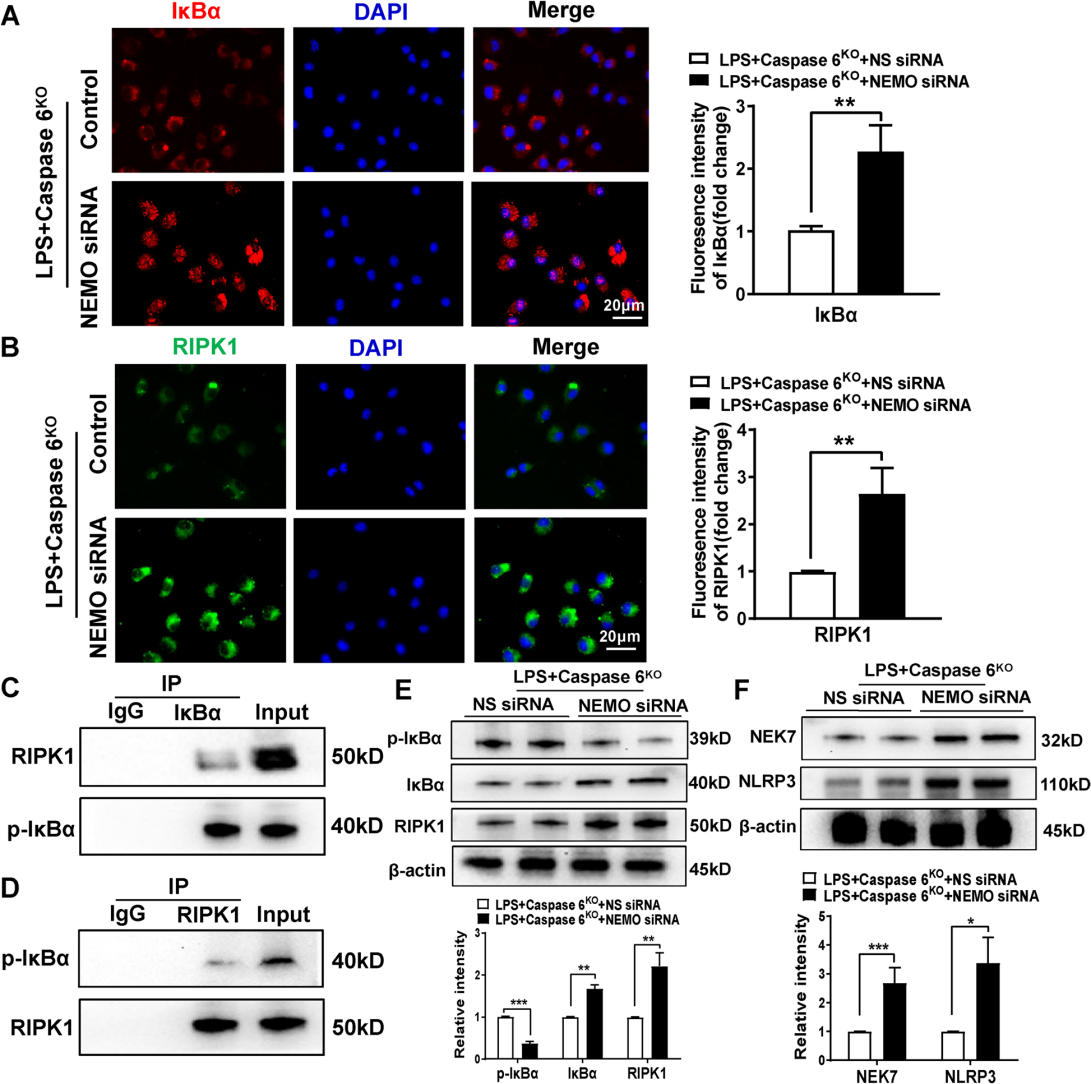
NEMO mediated RIPK1-IκBα interaction induces RIPK1 degradation while inhibited NEK7/NLRP3 function in macrophages. Bone marrow-derived macrophages (BMMs) derived from Caspase 6^KO^ mice were transfected with NEMO siRNA for 24 h followed by LPS treatment for 6 h. Immunofluorescence staining of IκBα (**A**) and RIPK1 (**B**) in LPS-stimulated macrophages. DAPI was used to visualize nuclei (blue). Scale bars: 20 μm, *N* = 4/group. **C**-**D** Immunoprecipitation analysis of RIPK1 and p-IκBα in LPS-stimulated macrophages from WT and Caspase 6.^KO^ mice, *N* = 5/group. **E** **F** Western blotting analysis and relative intensity of p-IκBα, IκBα, RIPK1, NEK7 and NLRP3, *N* = 6/group. All data represent the mean ± SD, *p < 0.05, **p < 0.01, ***p < 0.001

Having demonstrated that Caspase 6 deficiency activated NEMO/IκBα signaling and suppressed RIPK1 expression in ischemic livers, we proceeded to analyze putative interaction between RIPK1 and the NEMO/IκBα pathway in macrophages. Indeed, Western blot assay revealed that NEMO disruption inhibited the phosphorylation and subsequent degradation of IκBα while augmenting RIPK1 expression in mice ischemic livers (Fig. 5E). This finding was further confirmed by immunofluorescence staining of cultured macrophages, revealing enhanced IκBα and increased RIPK1 expression in BMMs from Caspase 6^KO^ mice after LPS stimulation (Fig. 5A-B). Strikingly, co-immunoprecipitation assay unveiled the co-localization of RIPK1 and p-IκBα in macrophage cytoplasm (Fig. 5C-D). Additionally, NEK7/NLRP3 activation was accelerated in Caspase 6-deficient BMMs pretreated with NEMO siRNA (Fig. 5F). Taken together, these data indicate that NEMO/IκBα-mediated RIPK1 degradation is required for the regulation of NEK7/NLRP3 function in Caspase 6-deficient ischemic livers.

### RIPK1/ASK1 axis promotes NEK7/NLRP3 inflammasome formation in Caspase 6-mediated immune response in macrophages

**Fig. 6:**
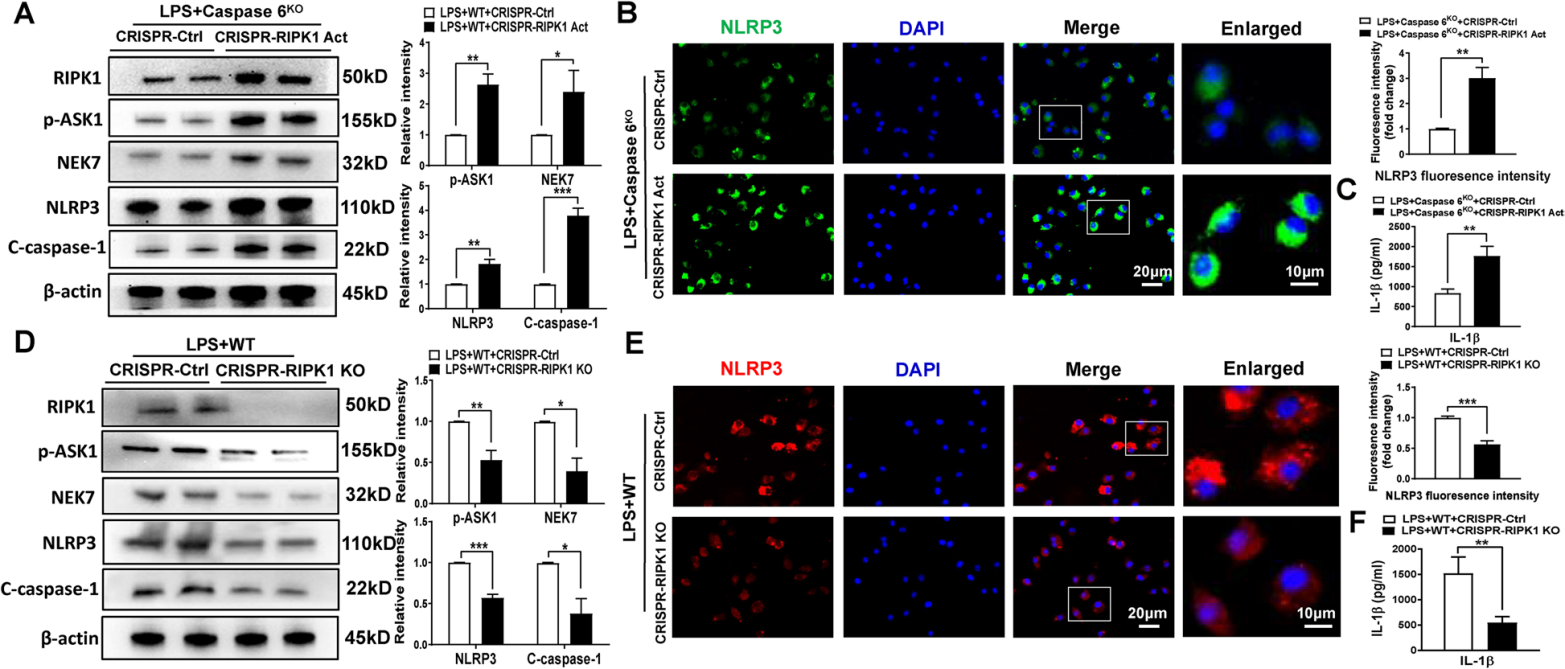
RIPK1-mediated ASK1 activation promotes NEK7/NLRP3 activation in Caspase 6-controlled inflammatory response in macrophages. BMMs were isolated from Caspase 6.^KO^ and WT mice and transfected with the p-CRISPR-RIPK1 activation, p-CRISPR-RIPK1 KO or control vector followed by LPS stimulation. **A D** Western blotting and relative intensity of RIPK1, p-ASK1, NEK7, NLRP3, and C-caspase-1, *N* = 6/group. **B E** Immunofluorescence staining for the NLRP3 expression in macrophages. DAPI was used to visualize nuclei. Scale bars: 20 μm, 10 μm, *N* = 4/group. **C F** ELISA analysis of IL-1β levels in culture medium, *N* = 6/group. All data represent the mean ± SD, *p < 0.05, **p < 0.01, ***p < 0.001

To further elucidate whether macrophage RIPK1 was involved in the NEK7/NLRP3-driven inflammatory response, BMMs isolated from Caspase 6^KO^ and WT mice were transfected with the p-CRISPR-RIPK1 activation, p-CRISPR-RIPK1 KO or control vector followed by LPS stimulation. Indeed, p-CRISPR-RIPK1 upregulated ASK1 phosphorylation, NEK7, NLRP3, and C-caspase-1 levels in LPS-stimulated BMMs isolated from Caspase 6^KO^ mice (Fig. 6A). Immunofluorescence staining revealed that RIPK1 activation aggravated NLRP3 expression in macrophages and IL-1β release (Fig. 6B-C). Conversely, CRISPR-RIPK1 KO vector curtailed ASK1 phosphorylation, NEK7/NLRP3 activation and IL-1β liberation from macrophages after LPS stimulation (Fig. 6D-F). Our results suggest that Caspase 6-related NEK7/NLRP3 function depends on the activation of RIPK1/ASK1 axis.

### Macrophage Caspase 6 signaling-mediated ASK1 activation regulates hepatocyte ferroptosis during inflammatory action

**Fig. 7:**
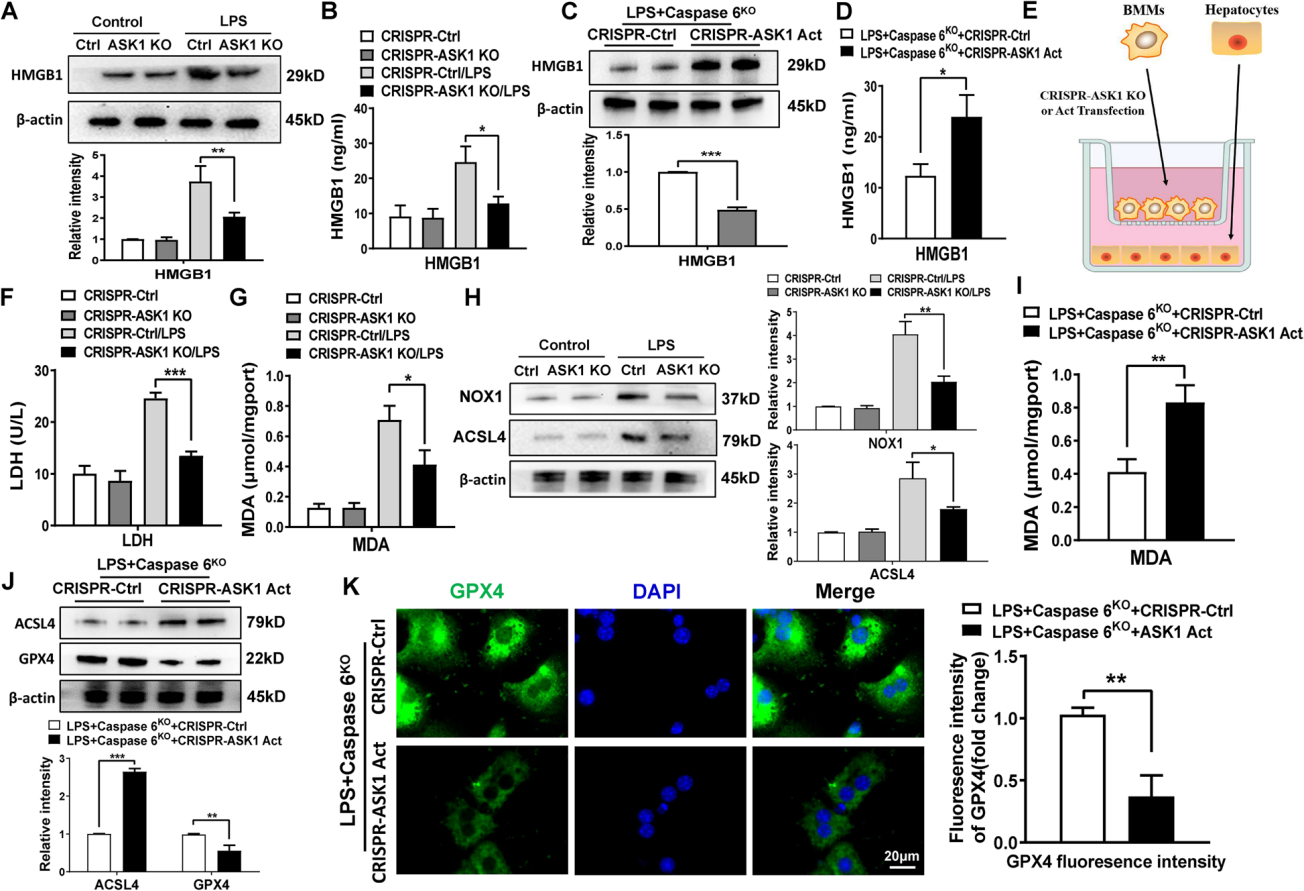
Macrophage Caspase 6 signaling-mediated ASK1 activation regulates hepatocyte ferroptosis during inflammatory action. BMMs were transfected with the p-CRISPR-ASK1 KO or control vector followed by LPS stimulation; (**A**) Western blotting and relative intensity of HMGB1 in LPS-stimulated BMMs, *N* = 6/group. **B** ELISA analysis of HMGB1 secretion in culture medium, *N* = 6/group. **C** Western blotting analysis of HMGB1 in ASK1 activated BMMs isolated from Caspase 6.^KO^ mice after LPS stimulation, *N* = 6/group. **D** HMGB1 release from hepatocytes, *N* = 6/group. **E** Schematic figure for macrophage/hepatocyte co-culture system; (**F**-**G**) LDH release and MDA concentration of hepatocytes in co-cultures, *N* = 5/group. **H** Western-assisted analysis and relative intensity of NOX1 and GPX4 in hepatocytes after co-culture, *N* = 6/group. **I** BMMs from Caspase 6-KO mice were transfected with the p-CRISPR-ASK1 Act or control vector followed by LPS stimulation. MDA concentration in hepatocytes was detected, *N* = 6/group. Ferroptosis-related proteins were measured in hepatocytes after co-culture by using western blot assay, *N* = 6/group (**J**) and Immunofluorescence staining, *N* = 4/group (K). All data represent the mean ± SD, *p < 0.05, **p < 0.01, ***p < 0.001

Considering HMGB1 is a critical regulator in ferroptosis priming [19], we queried whether ASK1 might influence hepatocytes ferroptosis during inflammatory response. We found that transfection with a p-CRISPR-ASK1 KO vector significantly decreased HMGB1 production and liberation from macrophages after LPS stimulation (Fig. 7A-B). In contrast, elevated HMGB1 release was observed in the p-CRISPR-ASK1 Act cells but not in the control cells (Fig. 7C-D). By using the co-culture system with LPS-stimulated BMMs and primary hepatocytes supplemented with H_2_O_2_ (Fig. 7E), we found that macrophage ASK1 deficiency markedly decreased LDH release and MDA concentration in stressed hepatocytes (Fig. 7F-G). Strikingly, unlike the control groups, p-CRISPR-ASK1 KO inhibited hepatocyte NOX1 and ACSL4 protein levels (Fig. 7H). Furthermore, in a co-culture system with LPS-stimulated p-CRISPR-ASK1 Act BMMs from Caspase 6^KO^ mice and hepatocytes, up-regulated MDA production (Fig. 7I) and ferroptosis-related proteins (Fig. 7J) were detected in hepatocytes. This was further substantiated by immunofluorescence staining, which showed the decreased expression of hepatocyte GPX4 after co-culture with the p-CRISPR-ASK1 Act BMMs but not the control cells (Fig. 7K). Taken together, these results indicate that macrophage Caspase 6 signaling-mediated ASK1 activation is the key to regulate hepatocyte ferroptosis during liver inflammatory action.

## Discuss

To the best of our knowledge, this study is the first study to establish an association between Caspase 6 expression and clinical prognosis in human livers after IR. Using both in vivo and in vitro parallel studies, we discover that hepatic IR activates Caspase 6 in macrophages, driving hepatic inflammatory responses through regulating RIPK1/IκBα-mediated innate immune responses. The principal findings are: (1) Elevated expression of Caspase 6 correlates with severe liver histopathology and functional injury in patients undergoing hepatectomy; (2) Caspase 6 deficiency reduces liver inflammation, oxidative stress and iron overload in ischemic livers; (3) Caspase 6 deficiency promotes NEMO-mediated phosphorylation of IκBα, causing p-IκBα to bind to RIPK1 and the subsequent RIPK1 degradation; (4) RIPK1-mediated ASK1 phosphorylation results in increased NEK7/NLRP3-driven liver inflammation and HMGB1-induced ferroptosis. Hence, this translational study provides extensive insight into the pivotal regulation of macrophage Caspase 6-mediated RIPK1-IκBα crosstalk in innate immune responses during hepatic IR.

Despite being categorized as an apoptotic executor and associated with neurodegenerative diseases [6, 20], the activation mechanism and biological function of Caspase 6 remain enigmatic. Active Caspase 6 has been postulated to induce cytochrome c release by cleaving Bid, leading to hepatocytes apoptosis [7]. Recent studies have demonstrated the non-apoptotic roles of Caspase 6. In the development of fracture-related pain, Caspase 6 was reported to regulate AMPA receptor trafficking [21]. In addition, Caspase 6-mediated p62 cleavage modulated autophagosome formation [22]. Despite these insights, the molecular mechanism of macrophage Caspase 6 in innate immunity response is poorly understood. Endogenous Caspase 6 can be expressed in both human liver samples and mice liver tissues subjected to liver IR and showed a positive correlation with liver injury. Furthermore, IR stress enhanced the expression of Caspase 6 mainly in hepatic macrophages but not hepatocytes, indicating the novel role of macrophage- derived Caspase 6 in acute liver inflammatory injury. To verify the underlying mechanism of Caspase 6, WT and Caspase 6^KO^ mice were assessed to investigate the degree of damage in ischemic liver. As expected, Caspase 6 deficiency markedly alleviated hepatic IR injury, as evidenced by reduced serum ALT levels, dampened iron overload, downregulated production of proinflammatory cytokines and oxidative stress mediators, and reduced CD11b + macrophage/Ly6G + neutrophil infiltration. The NLRP3 inflammasome is a multimeric component and activated in response to diverse cellular perturbations [23]. NEK7 is identified as an essential regulator during NLRP3 inflammasome assembly [24]. This assembly will result in the activation of Caspase 1 and the secretion of pro-inflammatory cytokines [25]. In line with previous findings, our data showed that Caspase 6 deficiency dampened NEK7, NLRP3 and cleaved-Caspase 1 expression, along with a reduction in IL-1β secretion. Thus, our data suggests that macrophage-derived Caspase 6 attenuates NEK7/NLRP3 inflammasome formation by inhibiting innate immune activation following hepatic IR.

Of particular interest, RIPK1 expression was significantly reduced in Caspase 6 deficient macrophages. RIPK1, a member of the serine-threonine kinases family, has emerged as a potential therapeutic target for diverse human diseases, including autoimmune diseases, cancer progression, inflammatory diseases and neurodegenerative diseases [26, 27]. A growing number of studies have revealed that RIPK1 is recognized as a central driver in both innate immune response and cellular stress response [28]. During early atherosclerotic progression in mice and humans, RIPK1 has been found to act as a pro-inflammatory hub in the vessel wall, resulting in persistent activation of proinflammatory mediators [29]. Notably, human RIPK1 deficiency has been linked to combined immunodeficiency and severe colitis caused by defective lymphocyte functions, enhanced inflammasome activity and altered response to TNF-α-mediated cell death [26]. Considering the kinase activity of RIPK1 is essential to innate immunity priming, the regulation of RIPK1 activation has been extensively investigated. Some evidence suggests that RIPK1 can be regulated by caspase mediated cleavage [30]. BJ van Raam et al. identified that endogenous Caspase 6 triggered apoptosis by cleaving RIPK1 in the apoptotic cascade [31]. However, the physiological relevance of the RIPK1 cleavage by caspases remains incompletely understood. Interestingly, our data contradicts previous studies which showed that lack of Caspase 6 leads to RIPK1 induction during embryonic development [31]. In our study, Caspase 6 deletion induced RIPK1 degradation in liver IR models. These different regulatory effects may be ascribable to varying pathophysiological conditions.

The question arises as to what the exact mechanisms may confer Caspase 6 with the ability to selectively guide RIPK1 signaling in the modulation of innate immunity priming. Our findings uncovered that the deletion of Caspase 6 downregulated RIPK1 expression while elevating NEMO and its downstream protein-phosphorylated IκBα levels in response to IR stimulation. NEMO is critical for canonical NF-κB-mediated signaling activation [32]. NEMO mutation is associated with the development of genetic diseases such as immunodeficiency, tumorigenesis, and pigmentary dysregulation [33]. Upon stimulation, NEMO becomes activated and facilitated either positive or negative regulation of the inhibitor of kB kinase (IKK) complex, which subsequently impacts the phosphorylation and consequent proteasomal degradation of IκBs. In mice and humans, three IκB subtypes (IκBα, IκBβ and IκBε) are involved in NF-κB retention in the cytoplasm. However, among those IκB subtypes, IκBα is the only essential gene for development as its absence leads to fatal death during perinatal period [34]. In mouse embryonic fibroblast, recombinant NEMO administration incited the phosphorylation of IκBα [35]. In this pathway, p-IκBα underwent ubiquitination and was rapidly degraded by the proteasome. This liberated NF-κB proteins and allowed their translocation to the nucleus [36]. Consistent with previous studies, our in vivo study showed that disruption of macrophage NEMO via mannose-mediated NEMO siRNA delivery system reversed the cytoprotection induced by Caspase 6 deficiency, as evidenced by exaggerated liver injury, augmented innate immunity and oxidative stress activation. Furthermore, our in vitro data found that macrophage NEMO knockdown diminished downstream p-IκBα but augmented RIPK1. Additionally, RIPK1 and IκBα co-localized in the cytoplasm of macrophages by using co-immunoprecipitation assay. the NEK7/NLRP3 inflammasome formation was also enhanced in NEMO-siRNA pretreated macrophages. Herein, the results above indicate that the interaction between RIPK1 and IκBα in macrophages is the necessary mediator for the modulation of Caspase 6-driven innate inflammation in ischemic livers.

ASK1 serves as a signaling node where diverse stressors congregate. Liver ASK1 may be a potential target to tackle non-alcoholic fatty liver disease and fibrosis [37]. Our previous study proved that ablation of myeloid Notch1 promoted ASK1 activation and hepatocellular apoptosis [17]. In our findings, increased ASK1 phosphorylation was observed in mice in response to IR stimulation. Further evidence demonstrated that RIPK1 activation in Caspase 6^KO^ macrophages augmented ASK1 activity and NEK7/NLRP3 function, suggesting that RIPK1/ASK1-mediated NLRP3 inflammasome activation is regulated by Caspase 6 signaling in ischemic livers. Hepatic macrophages are essential for initiating hepatic inflammatory response. Activation of macrophages can lead to the liberation of DAMPs from injured or inflamed cells, directly or indirectly predisposing to hepatocyte killing [3]. We have found that macrophage Caspase 6 deficiency might regulate liver ferroptosis, accompanied with an increase in serum HMGB1. Consistent with our in vivo results, ablation of macrophage ASK1 dampened the secretion of HMGB1, and reduced hepatocytes ferroptosis using macrophage/hepatocytes co-culture system. Additionally, ASK1 activation markedly exaggerated HMGB1 release from macrophages and induced ferroptosis activation of hepatocytes after co-culture with Caspase 6^KO^ macrophages.

Ferroptosis is now appreciated as one of the most widespread forms of cell death featured with iron overload and lipid oxidative damage [38]. Emerging evidence proves that ferroptosis is closely associated with multiple pathological processes, including tumor suppression, neurodegenerative diseases and immune surveillance [39, 40]. Numerous genes have been identified to regulate ferroptosis and serve as markers for ferroptosis. For instance, GPX4 and NOX1 function as negative regulators of ferroptosis by limiting ROS production and reducing iron uptake into cells. On the other hand, Transferrin receptor 1 and ACSL4 serve as positive regulators of ferroptosis by promoting ROS production and stimulating iron uptake [41]. In human and mice samples, iron accumulation and ferroptosis activation were observed and positively correlated with Caspase 6 expression. Further evidence was proved in vitro models, which showed that macrophage ASK1 triggered severe hepatocytes ferroptosis by releasing HMGB1. In conclusion, our findings affirm that Caspase 6-mediated RIPK1-IκBα interaction is a fundamental regulator of ASK1-mediated hepatocytes ferroptosis. Clearly, future in-depth assessment in the gene modification therapy targeting macrophage Caspase 6 is certainly warranted.

## Conclusion

This translational study elaborates a previous unrecognized role of macrophage-specific Caspase 6 in IR-stressed livers of both mice and humans. Mechanistically, Caspase 6 modulates functional interaction between macrophage RIPK1 and IκBα, thus contributing to hepatocytes ferroptosis during inflammation response. Macrophage Caspase 6 may serve as a biomarker for susceptibility to postoperative inflammation burst and the subsequent liver damage, thereby guiding early postoperative management for therapeutic intervention. Additionally, targeting Caspase 6 and downstream RIPK1-IκBα signals may provide new strategies for preventing liver sterile inflammatory injury.

### Supplementary Information

**Additional file 1.** 

**Additional file 2:** **Table S1.** Patient characteristics. **Table S2.** Primer sequences for the amplification (H, denotes human and M, denotes mice).

## Data Availability

The data used to support the findings of this study are available from the corresponding author upon reasonable request.

## Abbreviations

IR: Ischemia/reperfusion
Caspase: Cysteinyl aspartate specific proteinase
p-IκBα: Phosphorylated-inhibitor of NF-κBα
RIPK1: Receptor-interacting serine/ threonine-protein kinase 1
ASK1: Apoptosis signal-regulating kinase 1 ()
NEK7: NIMA-related kinase 7
NLRP3: NOD-like receptor family pyrin domain containing 3
DAMPs: Danger associated molecular patterns
HMGB1: High mobility group protein 1
AMPK: AMP-activated protein kinase
RIPK1: Receptor-interacting serine/threonine-protein kinase 1
TAK1: TGFβ activated kinase 1
ALT: Alanine aminotransferase
AST: Aspartate transaminase
KO: Knockout
WT: Wild-type
NS: Non-specific
MDA: Malonydialdehyde
IHC: Immunohistochemistry
NPCs: Nonparenchymal cells
PAGE: Polyacrylamide gel electrophoresis
SD: Standard deviation
ANOVA: One-way analysis of variance
NOX1: NADPH oxidase 1
ACSL4: Acyl-CoA synthetase long-chain familymember4
NEMO: NF-κB essential modulator
