# Supplementary figures and images for: Caspase 6 promotes innate immune activation by functional crosstalk between RIPK1-IκBα axis in liver inflammation
Source: Cell Commun Signal. 2023 Oct 12;21:282. doi: 10.1186/s12964-023-01287-x (PMC10568785; doi:10.1186/s12964-023-01287-x)

Fig .1H

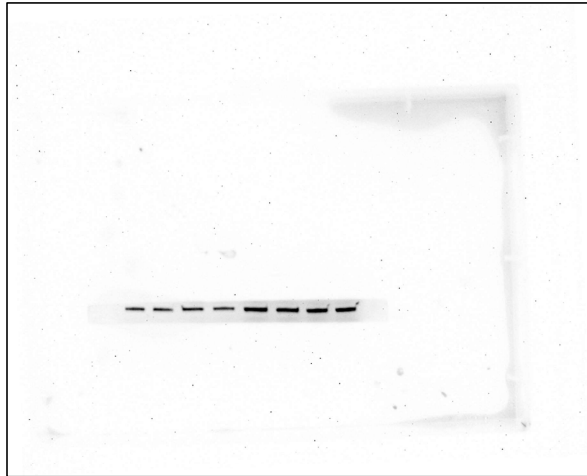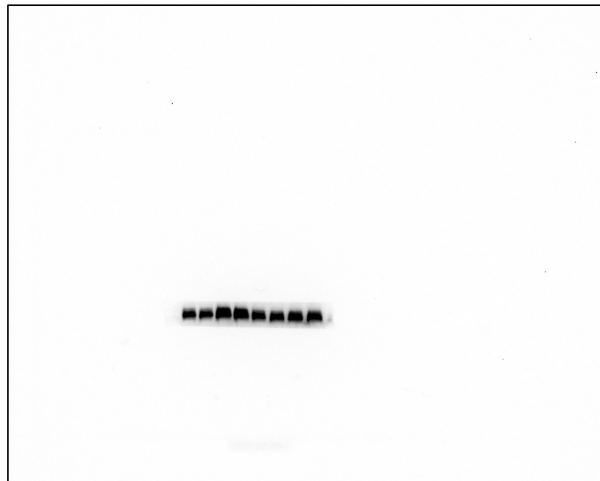

Fig .1I

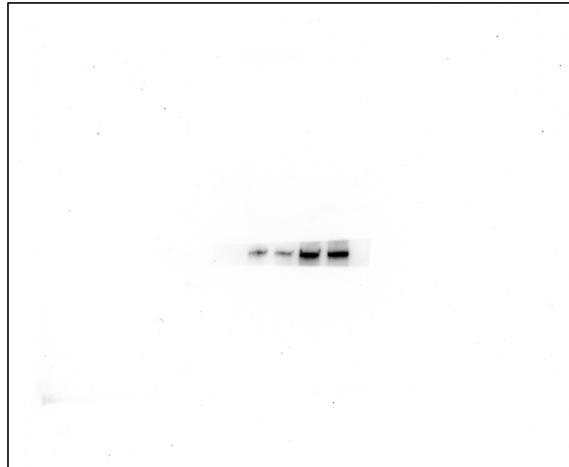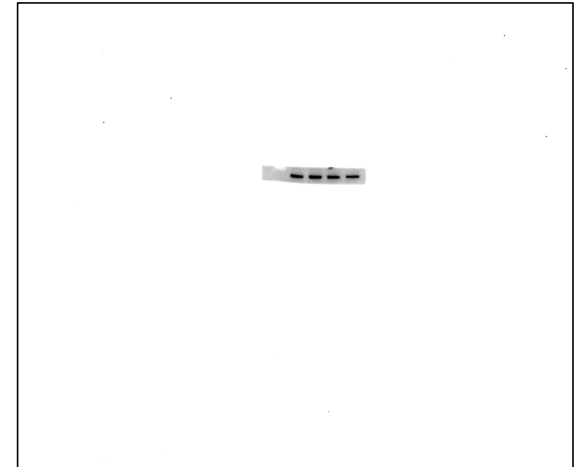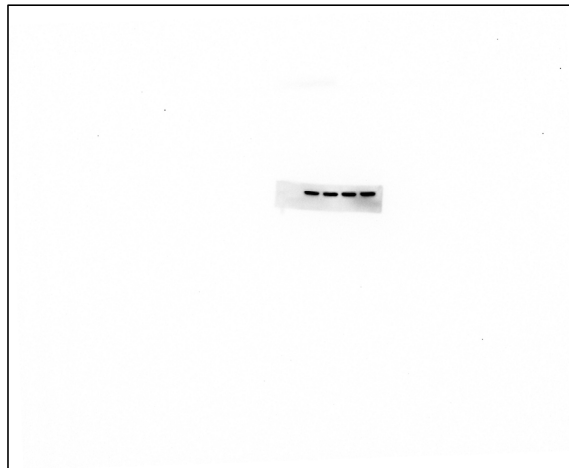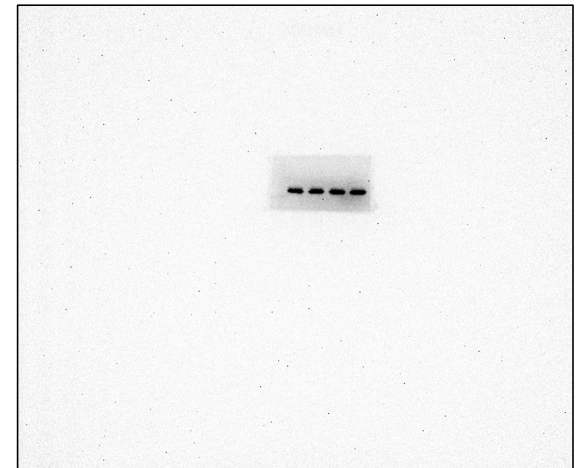

Fig .2A

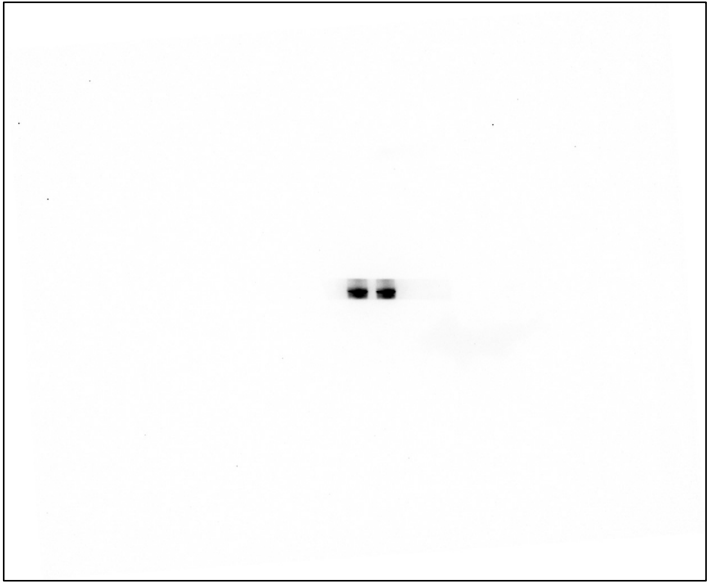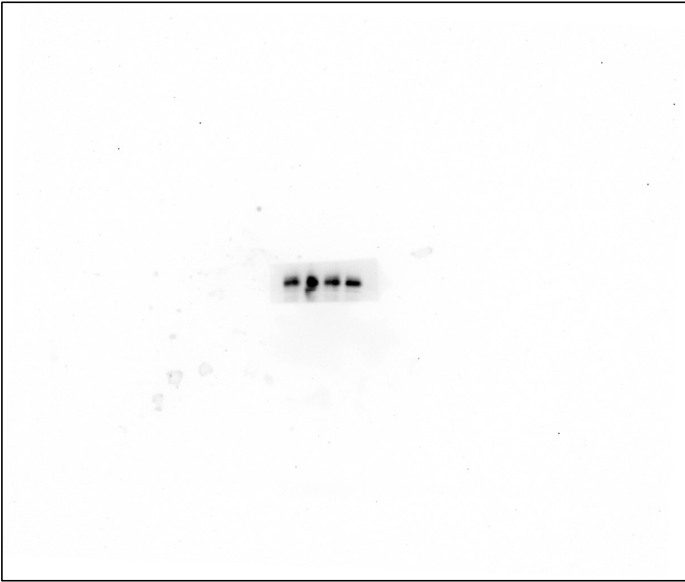

Fig .3A

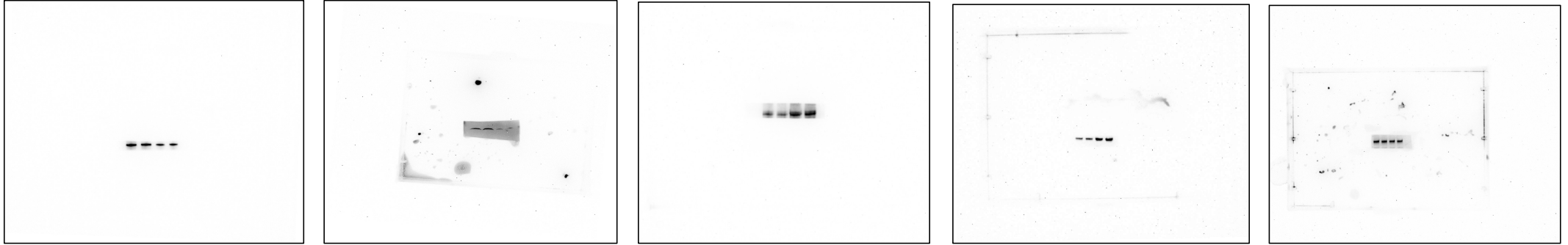

Fig .3B

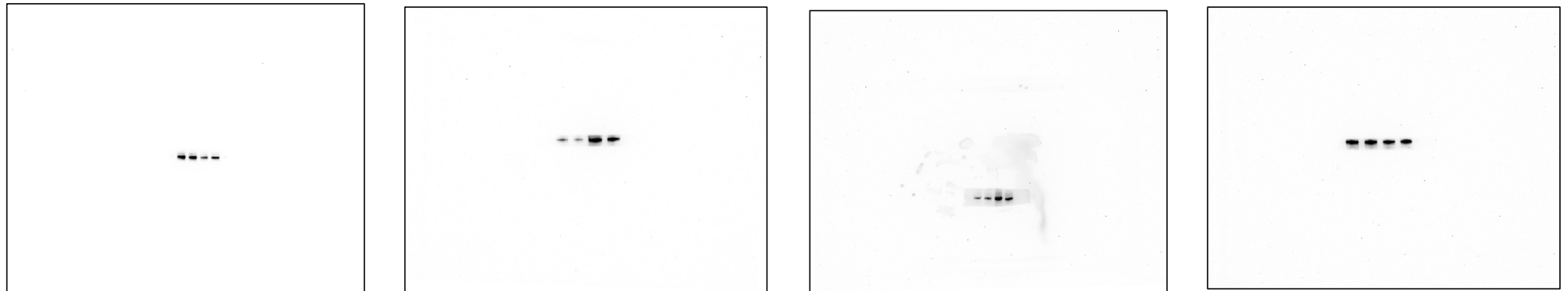

Fig .3C

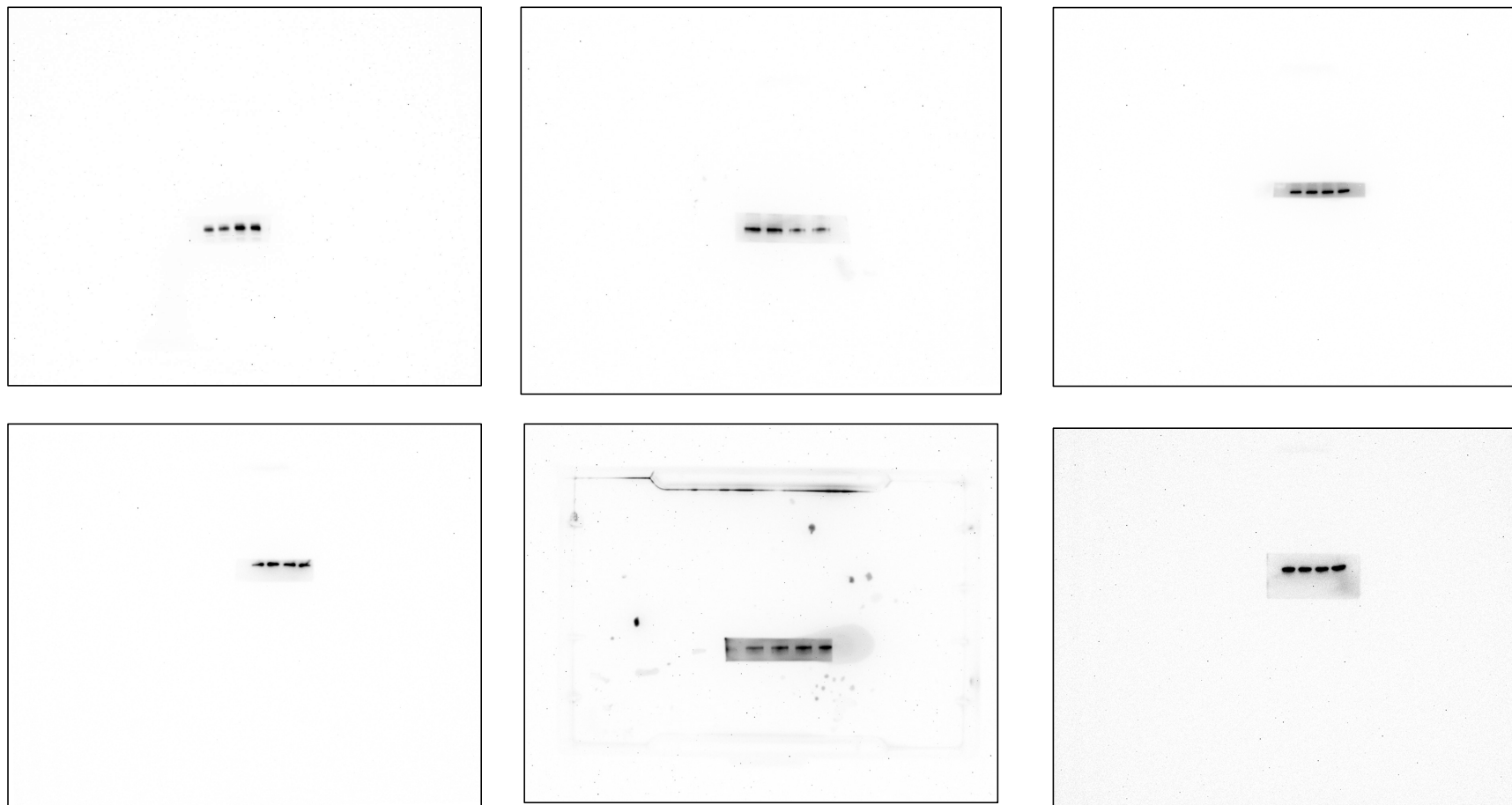

Fig .3D

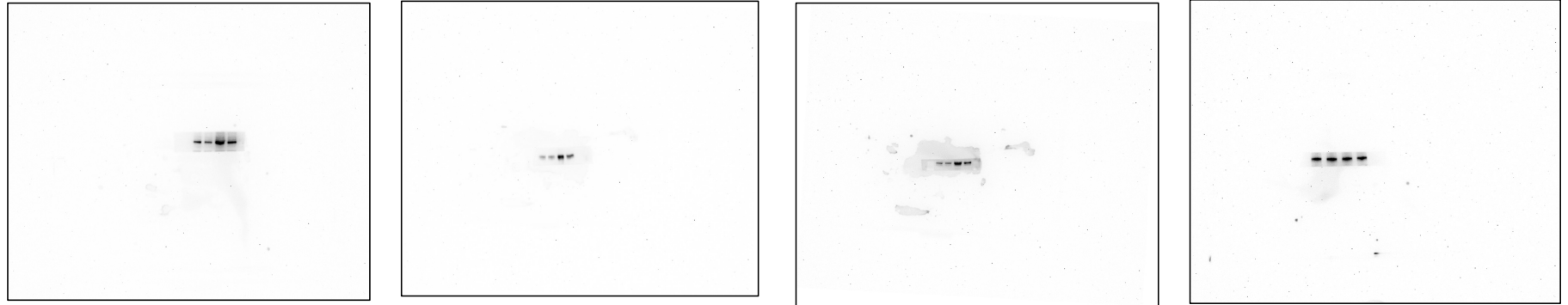

Fig .3F

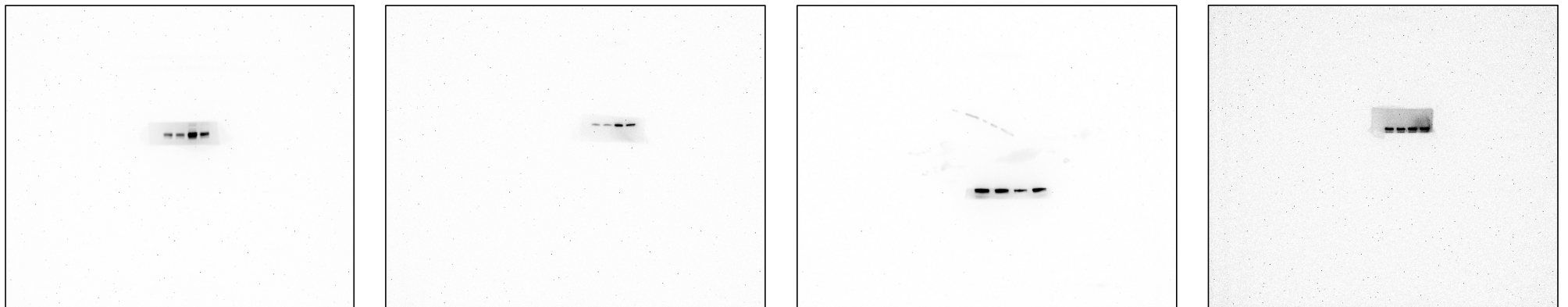

Fig .4C

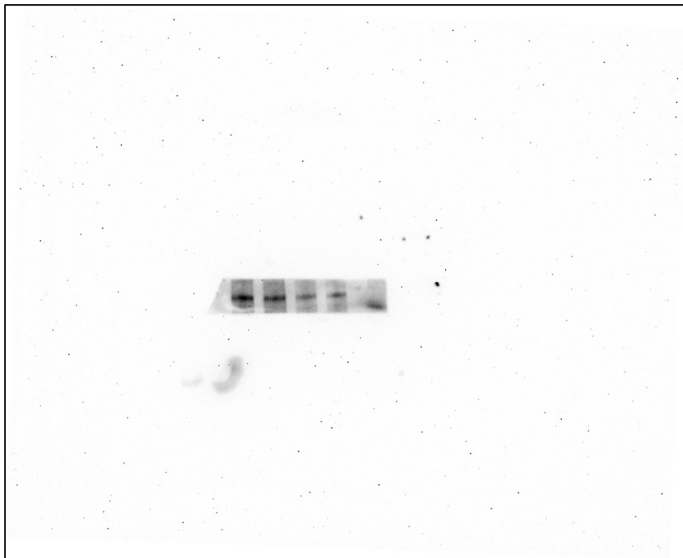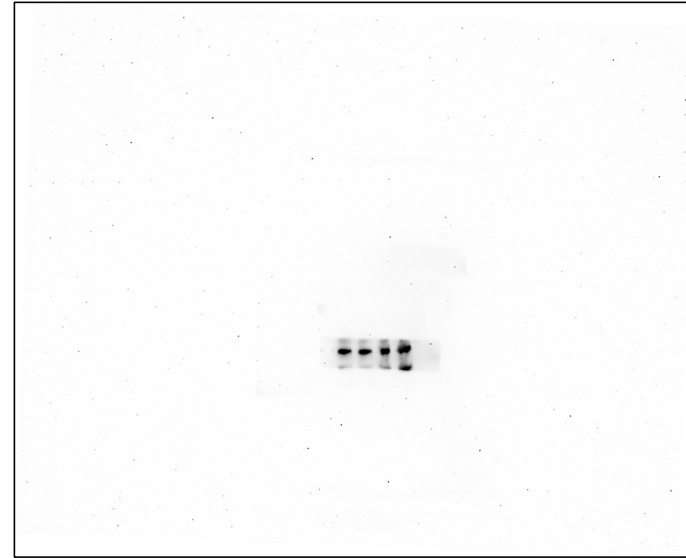

**Fig .5C**

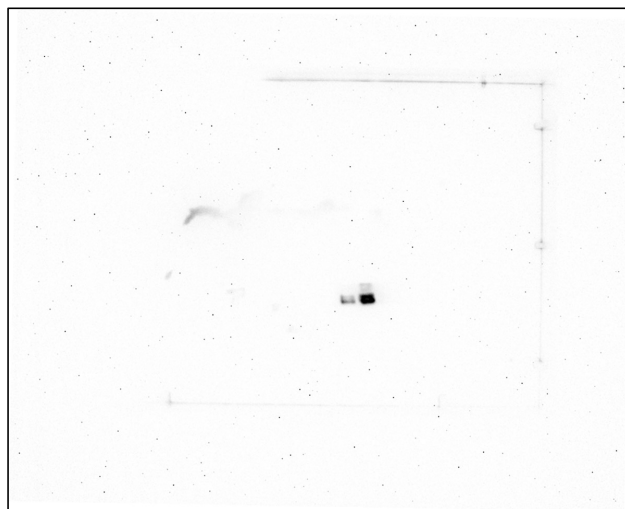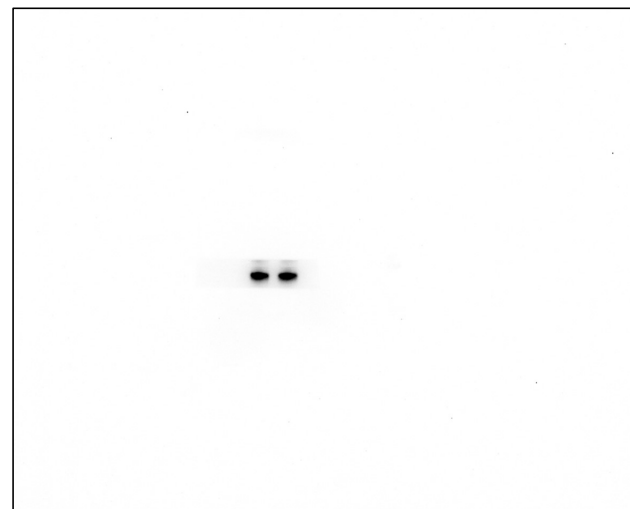

**Fig .5D**

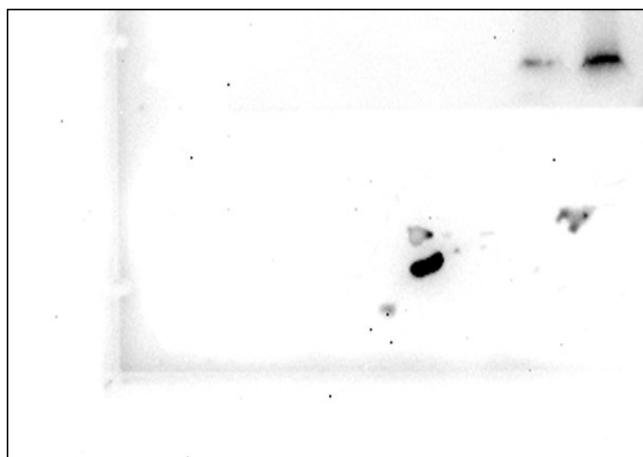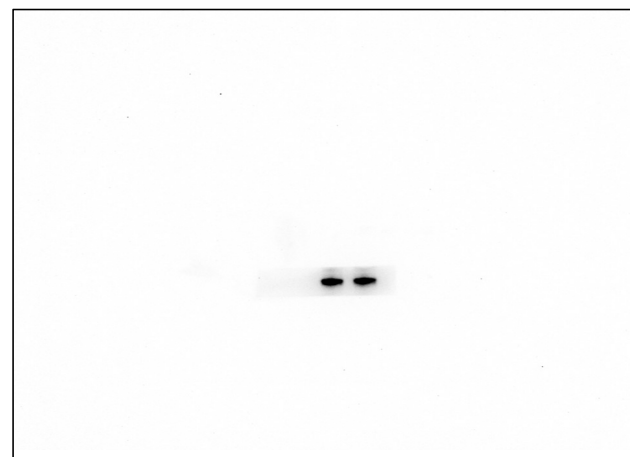

Fig .5E

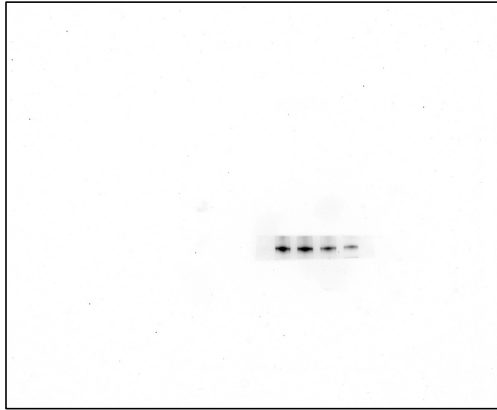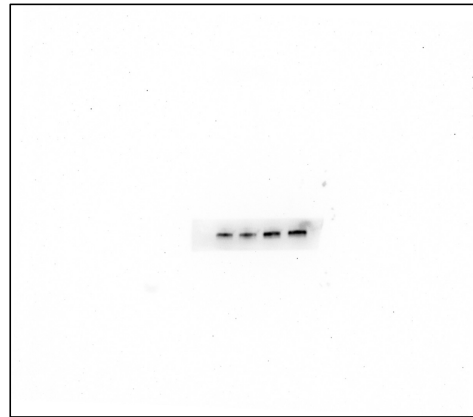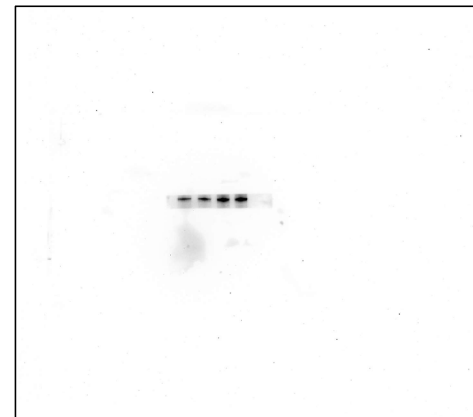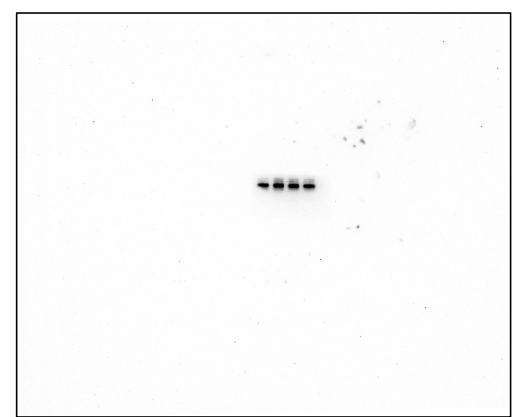

Fig .5F

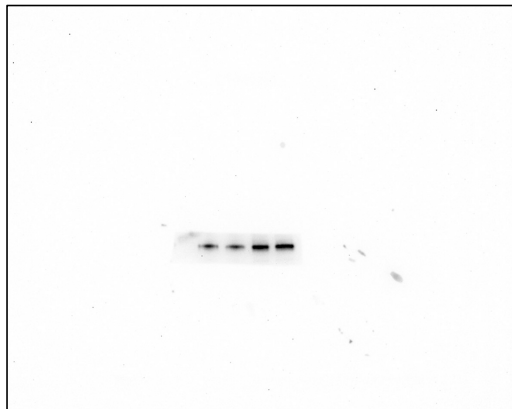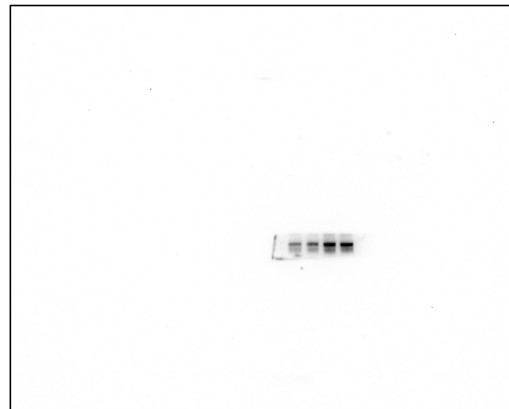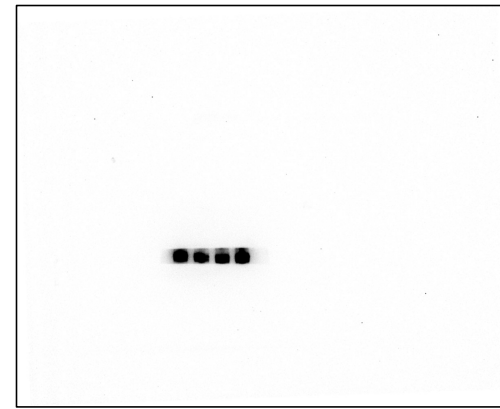

Fig .6A

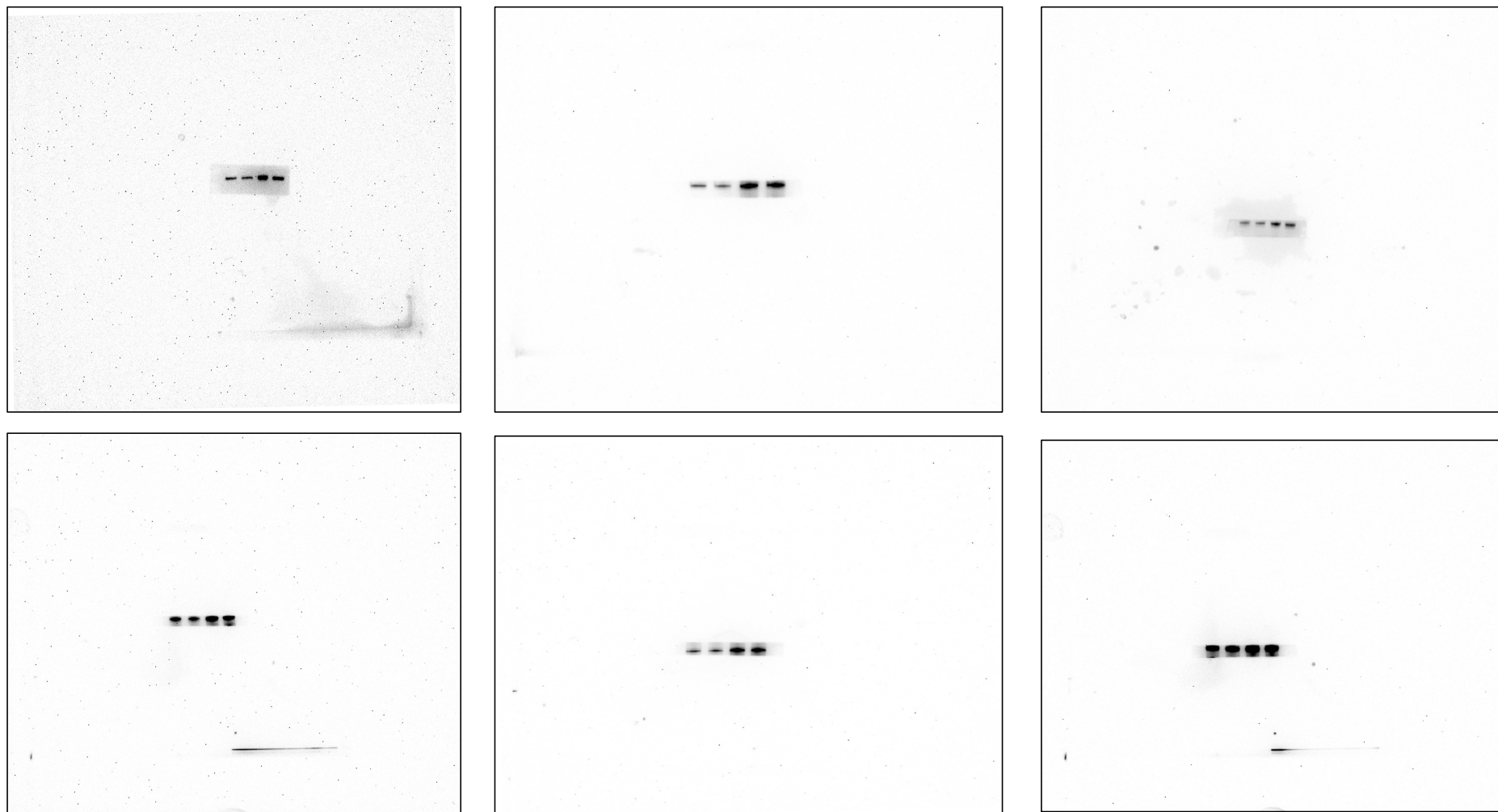

Fig .6D

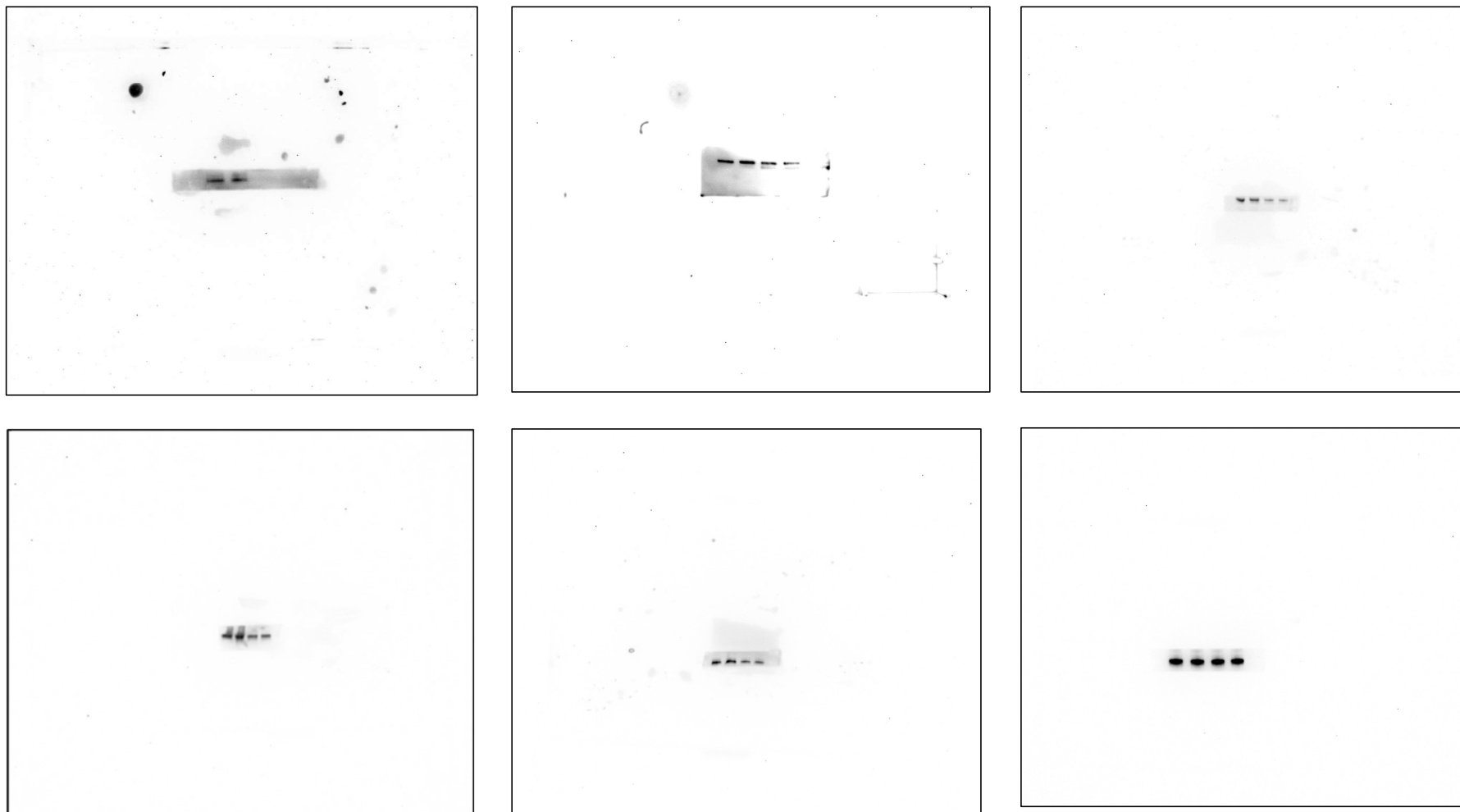

Fig .7A

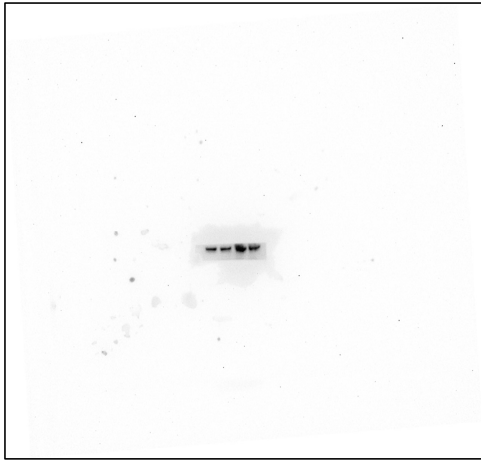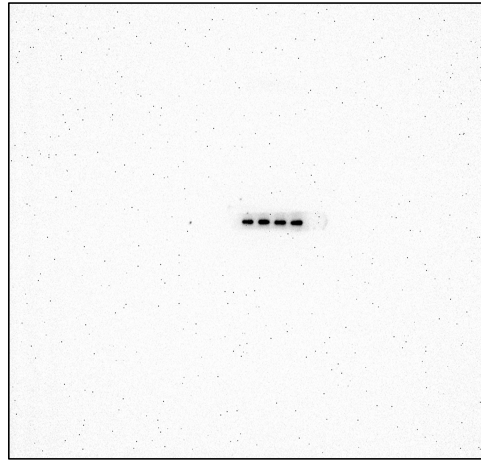

Fig .7C

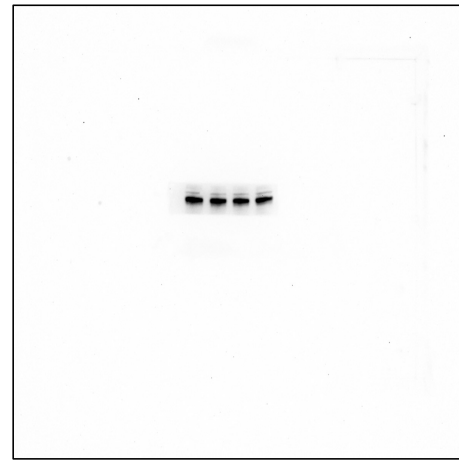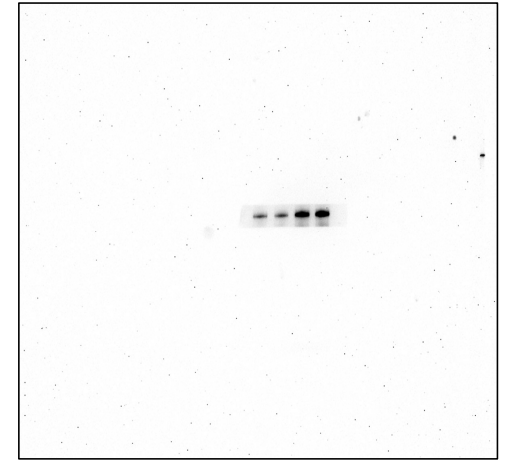

Fig .7H

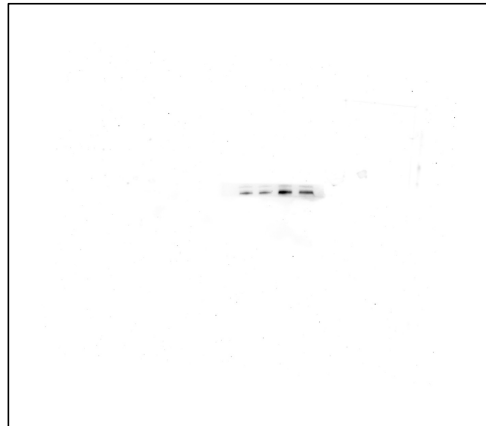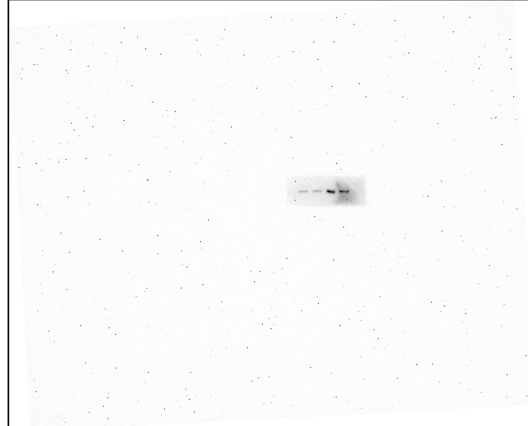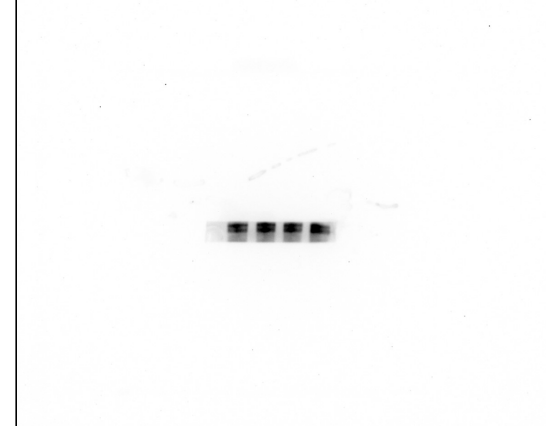

Supplement: Supplementary file 2 — Additional file 1. [file 12964_2023_1287_MOESM1_ESM.pdf]
