# Supplementary material for: Caspase 6 promotes innate immune activation by functional crosstalk between RIPK1-IκBα axis in liver inflammation
Source: Cell Commun Signal. 2023 Oct 12;21:282. doi: 10.1186/s12964-023-01287-x (PMC10568785; doi:10.1186/s12964-023-01287-x)
Supplement: Supplementary file 3 — Additional file 2: Table S1. Patient characteristics. Table S2. Primer sequences for the amplification (H, denotes human and M, denotes mice). [file 12964_2023_1287_MOESM2_ESM.docx]

**Supplementary Materials**

**Caspase 6 promotes innate immune activation by functional crosstalk between RIPK1-IκBα axis in liver inflammation**

Yuanbang Lin^1#^*****, Mingwei Sheng^2#^, Peng Zhang^1^, Chunli Wang^1^

^1^Department of General Surgery, Tianjin Medical University General Hospital, Tianjin, China.

^2^Department of Anesthesiology, Tianjin First Central Hospital, Tianjin, China.

^#^These authors contributed equally.

***Corresponding author**: Yuanbang Lin, MD, PhD., Department of General Surgery, Tianjin Medical University General Hospital, Anshan Road NO. 154, Tianjin, PR China, 300052. Email: linyuanbang@tmu.edu.cn.

**Table S1. Patient characteristics**

| **Clinical manifestation** | Patients with  high Caspase 6 | Patients with  low Caspase 6 |
| --- | --- | --- |
| Age, years  (mean ± SD) | 43.27 ± 5.37 | 49.01 ± 9.20 |
| Gender, n (M/F) | 4/11 | 6/9 |
| Etiology of disease (N) | Hepatic hemangioma (12)  Hepatic cyst (1)  Intrahepatic cholelithiasis (2) | Hepatic hemangioma (11)  Hepatic cyst (3)  Intrahepatic cholelithiasis (1) |
| **Laboratory tests**  (mean ± SD) |  |  |
| Serum ALT of POD1, U/L | 745.3 ± 48.59 | 389.1± 69.12 |
| Serum ALT of POD1, U/L | 611.4±302.9 | 420.44 ± 50.61 |

ALT, Alanine aminotransferase; AST, Aspartate aminotransferase; POD. the first days postoperatively.

**Table S2**: Primer sequences for the amplification (H, denotes human and M, denotes mice)

| **Target genes** | **Forward primers** | **Reverse primers** |
| --- | --- | --- |
| H-Caspase 6 | 5’-CGATGTGCCAGTCATTCCTT-3’ | 5’-CTCTAAGGAGGAGCCATAT-3’ |
| H-β-actin | 5’-TAAGGAGAAGCTGTGCTACGTC-3’ | 5’-AGTTTCGTGGATGCCACAGG-3’ |
| M-Caspase 6 | 5’-AAGTGTTCGATCCAGCCGAG-3’ | 5’- CAGGTTGTCTCTGTCTGCGT-3’ |
| M-IL-1β | 5’- TGTAATGAAAGACGGCACACC-3’ | 5’-TCTTCTTTGGGTATTGCTTGG-3’ |
| M-TNF-α | 5’-GCTACCAAACTGGATATAATCAGGA-3’ | 5’-CCAGGTAGCTATGGTACTCCAGAA-3’ |
| M-CXCL-10 | 5’-GCTGCCGTCATTTTCTGC-3’ | 5’-TCTCACTGGCCCGTCATC-3’ |
| M-β-actin | 5’-GTGACGTTGACATCCGTAAAGA-3’ | 5’-GCCGGACTCATCGTACTCC-3’ |
